# Development of PCR, LAMP and qPCR Assays for the Detection of Aflatoxigenic Strains of *Aspergillus* *flavus* and *A. parasiticus* in Hazelnut

**DOI:** 10.3390/toxins12120757

**Published:** 2020-11-30

**Authors:** Sara Franco Ortega, Ilenia Siciliano, Simona Prencipe, Maria Lodovica Gullino, Davide Spadaro

**Affiliations:** 1 Centre of Competence for the Innovation in the Agro-Environmental Sector—AGROINNOVA, University of Turin, via Paolo Braccini 2, I-10095 Grugliasco, Italy; sarafrancoortega@gmail.com (S.F.O.); Ilenia.siciliano@unito.it (I.S.); marialodovica.gullino@unito.it (M.L.G.); 2 Department of Agricultural, Forestry and Food Sciences (DiSAFA), University of Torino, via Paolo Braccini 2, I-10095 Grugliasco, Italy; simona.prencipe@unito.it

**Keywords:** LAMP, qPCR, detection, hazelnuts, *Aspergillus flavus*, *A. parasiticus*, aflatoxins

## Abstract

*Aspergillus flavus* and *A. parasiticus* are two species able to produce aflatoxins in foodstuffs, and in particular in hazelnuts, at harvest and during postharvest phase. As not all the strains of these species are aflatoxin producers, it is necessary to develop techniques that can detect aflatoxigenic from not aflatoxigenic strains. Two assays, a LAMP (loop-mediated isothermal amplification) and a real time PCR with TaqMan^®^ probe were designed and validated in terms of specificity, sensitivity, reproducibility, and repeatability. The capability of the strains to produce aflatoxins was measured in vitro and both assays showed to be specific for the aflatoxigenic strains of *A. flavus* and *A. parasiticus*. The limit of detection of the LAMP assay was 100–999 picograms of DNA, while the qPCR detected 160 femtograms of DNA in hazelnuts. Both techniques were validated using artificially inoculated hazelnuts and naturally infected hazelnuts. The qPCR was able to detect as few as eight cells of aflatoxigenic *Aspergillus* in naturally infected hazelnut. The combination of the LAMP assay, which can be performed in less than an hour, as screening method, with the high sensitivity of the qPCR, as confirmation assay, is able to detect aflatoxigenic strains already in field, helping to preserve the food safety of hazelnuts.

## 1. Introduction

Aflatoxins (AFs) are toxic polyketide secondary metabolites with low molecular weight and hepatocarcinogenic activity. These compounds are poorly soluble in water and highly soluble in moderately polar solvents, such as chloroform, methanol, or dimethylsulfoxide. Regarding their chemical structure, AFs can be divided into two groups identified based on the fluorescence emitted under ultraviolet light: type B (blue fluorescence) with a difuro-coumaro-cyclopentenone structure; type G (green fluorescence) with a six-member lactone ring replacing the cyclopentenone. Eighteen different types of AFs are currently identified [1], with the most important and common aflatoxins in nature being aflatoxin B1 (AFB1), B2 (AFB2), G1 (AFG1), and G2 (AFG2).

*Aspergillus* section *Flavi* includes 33 species and most of them are aflatoxin producers, such as *Aspergillus flavus* Link, *A. parasiticus* Speare, and *A. toxicarius* Murak [2,3,4]. However, non-aflatoxigenic species have also been identified, such as *A. oryzae*, *A. sojae*, and *A. tamarii*. Some of the non-aflatoxigenic species, such as *A. oryzae* and *A. sojae*, are widely used in the oriental food fermentation process, being considered as the domesticated species of *A. flavus* and *A. parasiticus* [5,6]. *A. flavus* is the main producer of AFB1, AFB2, and cyclopiazonic acid (CPA) [7,8] while the closely related species *A. parasiticus* and *A. toxicarius* are important producers of AFB1, AFB2, AFG1 and AFG2. Though, not all *A. flavus* strains are aflatoxigenic, depending on the functionality or not of the entire aflatoxin biosynthesis gene cluster [9].

Hazelnut (*Corylus avelana* L.) is an important product for human nutrition that has beneficial effects on human health by reducing cholesterol and preventing of chronic diseases, such as cancers or cardiovascular complications [10], due to the high concentration of monounsaturated fatty acids, especially oleic acid, and polyunsaturated fatty acids [11]. Italy is the second biggest world producer country of hazelnuts after Turkey. Production of hazelnuts in Italy was 132,699 tons in 2018 [12], representing 13% of the worldwide production [13].

Hazelnuts can be contaminated by different aflatoxigenic molds species at harvest, during storage and processing leading to significant economic losses and possible aflatoxins contamination. The harvest time and method significantly impact the presence of *Aspergillus* spp. on hazelnuts and therefore in the AFs content of the product. In Turkey, hazelnuts can be directly collected from the tree, but it is a time-consuming procedure and the most common harvest technique is the collection of the mature hazelnuts after dropping to the ground, which usually increases the mold contamination on the surface [14]. Traditionally, after harvesting, hazelnuts are sun-dried to decrease the water activity and to avoid mold growth. However, drying during rainy periods may also enhance the risk of aflatoxin contamination [14]. Hazelnuts storage before drying is another relevant factor, as the common practice of using nylon bags, which increase the humidity, moisten the hazelnuts and boost the susceptibility to fungal growth [14].

During postharvest, *Aspergillus* spp. and *Penicillium* spp., which are present at low levels in preharvest, become a threat in nuts, including hazelnuts, due to their adaptation to the storage conditions [14,15]. During postharvest storage, dried hazelnuts require low relative humidity and low temperature (5 °C and 65.5% RH), but when these conditions are not met, the risk of fungal contamination of the kernels and aflatoxin content can increase again [13,15,16,17,18].

Effective controls and accurate and efficient diagnosis should be developed to monitor and control *Aspergillus* spp. contaminations. The prediction of risk levels associated with mycotoxins in food and the compliance with legal limits and health regulations could be improved by the development of a Decision Support Systems (DSS) [19]. In this perspective, assessing the presence of mycotoxins and quantifying them through the different stages of production is essential. The EU has recently established the maximum level of AFs in nuts, including almonds, pistachios, and apricot kernels and hazelnuts [20]. The maximum level of AFB1 and total AFs in hazelnuts subjected to physical treatments is 8 µg/kg and 15 µg/kg, respectively, whilst it is 5 µg/kg for AFB1 and 10 µg/kg for total AFs in hazelnuts for human consumption. In the USA, the FDA has set 20 ng/g as the maximum level of AFs for nuts, whilst Japan has established the same levels of the EU for imported nuts used for direct consumption or as food ingredients [21,22].

The detection of *Aspergillus* spp. on food has been traditionally based on plate count and culturing methods studying macro- and micro-morphological characteristics. Traditional detection techniques are time-consuming and require well-trained staff. Molecular techniques overcome the risk of misidentification. Many attempts to develop the most specific and sensitive techniques, able to detect and quantify *Aspergillus* spp., have been performed. PCR-based assays have been developed to distinguish between aflatoxin producer and non-producer *Aspergillus* spp. based on the detection of several genes of the aflatoxin biosynthesis gene cluster, by using RNA-based assays, end-point PCR or PCR variants [23,24,25,26,27,28,29,30]. The assays developed included multiplex PCR [31], quantitative real time (qPCR) for the *Aspergillus* genus, *Aspergillus* section *Flavi* spp. [6,32,33,34], specific for *A. flavus* [35,36,37], and assays able to distinguish between *A. flavus* and *A. parasiticus* [38]. The loop-mediated isothermal amplification (LAMP) assay [39,40] has been confirmed as a robust method to detect plant pathogens as viruses, bacteria and fungi [41,42,43]. The high specificity of these assays is based on the use of six primers that recognize eight genetic regions in order to amplify a target DNA. More recently, LAMP assays have been designed to differentiate between ochratoxigenic *Aspergillus* spp. or aflatoxigenic and non-aflatoxigenic, or fumonisin producer strains of *A. flavus* [44,45,46,47,48,49]. In addition, a novel LAMP assay designed by Niessen et al. [50] was developed on the *nor1* gene for the detection of aflatoxin producers within *Aspergillus* section *Flavi*, showing high sensitivity and specificity. This methodology is relatively rapid and may compete with the traditional identification because of its specificity and sensitivity. The detection of the LAMP products can be based on the use of indicators such as calcein or neutral red which causes a color change of the reaction from yellow (negative) to pink (positive) [50]. However, colorimetric changes, such as the Niessen et al. [50] assay, can be difficult to interpret, especially for color-blind people or when the light conditions are not optimal (as in the field or inside storage areas).

As the LAMP assay is less sensitive to inhibitors, crude DNA extraction methods can be associated, which can speed up the DNA extraction from hours to minutes and in combination with portable devices, such as the Genie II and III (Optigene, Horsham, UK), which measure the fluorescence of the LAMP products, this assay can be quickly transferred to the food sector.

On the other hand, quantitative PCR is a robust diagnostic tool due to its higher sensitivity which is critical to detect samples with low target concentrations. Developing and validating these two methods to detect aflatoxigenic molds is important to avoid contamination of AFs in the food processing sector. In this study, we developed three different DNA-based methods, an end-point PCR, a LAMP assay, and a qPCR assay, to detect the presence of two of the most common aflatoxigenic species belonging to *Aspergillus* section *Flavi* that are important contaminants in hazelnuts, *A. flavus* and *A. parasiticus*. The combination of these analyses in different scenarios may help in obtaining a successful reduction in aflatoxin contamination in the tree nuts industry. Previously, LAMP assays were developed to detect aflatoxigenic molds on nuts, without chemical confirmation on the strains. In this work, we developed LAMP and qPCR assays for the detection of aflatoxigenic strains of *A. flavus* and *A. parasiticus* on hazelnuts. The results were verified by HPLC-FLD method for aflatoxin production both in vitro and in vivo, to evaluate the matrix effect [51].

## 2. Results

### 2.1. Aflatoxin Analysis In Vitro

Ninety strains of *Aspergillus* section *Flavi* isolated from hazelnuts (50 strains) (Table 1) and chestnuts (40 strains) (Table 2) and 7 CBS reference strains (Table S1) were analyzed for aflatoxin production in vitro. The identification of hazelnuts isolates was performed by sequencing the calmodulin gene which was later used for inferring a maximum likelihood tree (Figure S1). This gene identified 42 of the 50 isolates from hazelnuts. As eight isolates presented a low sequence quality, they were not included in the phylogenetic tree. However, visual observation identified these eight isolates as part of *Aspergillus* section *Flavi.* Most strains (39 out of 50) were identified as *A. flavus*, and three strains (FS8, BS1 and BS8) as *A. parasiticus/A. sojae.* Based on micro- and macro-morphological observations, the strains FS8 and BS1 were identified as *A. parasiticus*, while the strain BS8 was *A. sojae.*

AFB1 was the most frequently produced mycotoxin, followed by AFB2. In chestnuts strains, the AFB1 was produced by 13 out of 30 (43.3%) of *A. flavus* and by the *A. parasiticus* and *A. toxicarius* strains, whilst the AFB1 was produced by 28 out of 50 hazelnut strains (56.0%, corresponding to four strains that were not identified by the calmodulin gene, 22 *A. flavus* and two *A. parasiticus*) and most of these 28 strains were also able to produce the AFB2 (44.0%, corresponding to three strains that were not identified with the calmodulin gene, 17 *A. flavus* and two *A. parasiticus*). AFG1 and AFG2 were produced by *A. parasiticus* and *A. toxicarius* strains. One strain of *A. sojae* from hazelnut (BS8) did not produce any aflatoxin [4].

Regarding the leaves, bracts and kernel isolates, out of the 26 strains from hazelnut leaves, 13 strains produced AFB1 (*A. flavus* and *A. parasiticus*), and 10 of them also produced AFB2, whilst 11 and 10 strains from bracts produced AFB1 and AFB2, respectively. However, only three and two out of eight strains from kernels produced AFB1 and AFB2, respectively. AFG1 and AFG2 were only detected in one strain from leaves (FS8) and two from bracts (BS1 and BS6).

The highest amount of mycotoxin was produced by the strains identified as *A. toxicarius* and *A. parasiticus* isolated from chestnuts and by the strains FS8 and BS1 (*A. parasiticus)* and strains BS6 and FV5 (*A. flavus*) isolated from hazelnuts.

The method used for the quantification was validated for linearity, limit of detection (LOD), limit of quantification (LOQ), and matrix effect (ME). The analytical method was tested in a range of concentrations from 0.1 up to 20 ppb. After extraction, all the samples were diluted to remain in the linearity range. ME was calculated for the four AFs in yeast extract-sucrose (YES) culture broth and the result was 59% for AFB1, 57% for AFB2, 74% for AFG1, and 57% for AFG2. In order to minimize the susceptibility related to the matrix effect, the quantification of the analytes was obtained through the calibration curves prepared in the matrix. Calculated LODs were 0.1, 0.2, 0.4, and 0.2 ppb and calculated LOQs were 0.5, 0.7, 1.2, and 0.8 ppb for AFB1, AFB2, AFG1, and AFG2, respectively.

### 2.2. Identification of the Region for LAMP and qPCR Primer Design. PCR Validation

The presence or absence (deletion) of a PCR product of approximately 308 bp obtained with the primers AfF and AfR, listed in Table 3, was checked by means of agarose gel. The primers aligned with the aflatoxin biosynthetic cluster of different species (Figure S2) corresponding with the end of the last exon (AfF) and the beginning of the last intron (AfR) of the aflatoxin efflux pump (*aflT*) gene, as shown in the Figure S3. The product was present in 48 strains and reference strains. The product was not present in *A. oryzae* var. *oryzae* CBS 46691, and *A. flavus* var. *columnaris* (CBS 485.65) (Figure S4). The amplification was unclear for the strain BS1. Comparing with the aflatoxin production, BS6 (*A flavus*), BS9 (*A. flavus*), AFLX1 (*A. flavus*), AFLX2 (*A. flavus*), AFSP2 (*A. flavus*), AFSP3 (*A. flavus*) did not present the band, but were able to produce at least one of the four aflatoxins studied. On the contrary, FP8 (*A. flavus*), BV10 (*A. flavus*), BS5 (*A. flavus*), NP1 (*A. flavus*), NP6 (*A. flavus*), AFCAL7 (*A. flavus*), AFLX7 (*A. flavus*) and AFLX10 (*A. flavus*) present the band but no aflatoxin was detected. The sensitivity of the technique was validated using a standard curve with the DNA of the *A. flavus* FS3 (Figure S5) starting from 110 ng/µL and reaching an LOD of 111 fg/µL.

### 2.3. LAMP Assay Validation

The six LAMP primers, designed on a region covering 186 bp (product size calculated using the *A. parasiticus* sequence of the aflatoxigenic biosynthetic cluster, GenBank accession number: AY371490) of the aflatoxin biosynthetic gene cluster (Figure S2), are listed in Table 3. The primers aligned with the end of the last exon and the beginning of the last intron of the *aflT* gene as indicated in the Figure S3. Positive results of the LAMP assays were obtained only for the strains with the presence of a 308 bp PCR product checked by means of agarose gel. BLASTn analysis of F3, loopF, loopB, and FIP primers showed similarity at 100% with *A. flavus* NRRL 3357 (GenBank accession number CP0044620 and XM 002379911) that causes aflatoxin contamination on food and feed [48] and with the strains *A. flavus* AF36, *A. flavus* AF13, *A. flavus* BN008, *A. flavus* AF70, and other *A. parasiticus* and *A. flavus* strains (GenBank accession numbers: AY510455, AY510451, AY510452, AY510453, AY371490, AF515601, respectively), as well, they showed 100% similarity with *Aspergillus pseudotamarii* (GenBank accession number XM_032052844.1) and *A. sojae* (CP035528.1). BIP and B3 primers showed 100% similarity with *A. flavus* NRRL 3357, *A. flavus* AF36, *A. flavus* AF13, *A. flavus* AF70, and *A. parasiticus* and *A. flavus* (GenBank accession numbers: CP0044620.1, AY510455, AY510451, AY510452, AY510453, CP051029.1, AF515601, respectively) and approximately at 90% with *A. flavus* BN008 (AY510452.1). AF36 strain was reported as non-aflatoxigenic whilst the strain BN008 was reported to produce both B and G AFs by Ehrlich et al. [49], which is a common feature of *A. parasiticus*. According to the online software, the primers showed appropriate structural features, which avoid potential secondary structures. In order to verify the specificity of the LAMP primers, the PCR product obtained with the primers AfF and AfR using the DNA of FP8, BV10 and BS5 was sequenced, and the LAMP primers were found in the sequence, with all the primers matching at 100%.The specificity of the manually design assay was validated according to a panel of inclusion and exclusion of 90 *Aspergillus* spp. isolated from hazelnuts and chestnuts and seven CBS references (Table 1 and Table 2 and Table S1). Other common pathogens of hazelnuts and chestnuts were included in the panel (Table S2). Considering the 97 *Aspergillus* spp., and the in vitro mycotoxin production, the LAMP assay produced 42 real positives including the species *A. subolivaceus* CBS 501.65, *A. thomii* CBS 120.51, *A. arachidicola* CBS 117610, *A. minisclerotigenes* CBS 117635 and *A. parvisclerotigenus* CBS 121.62, 2 false positives, 5 false negatives and 48 real negatives. Therefore, no LAMP amplification was found in most of the non-aflatoxin producer strains and nor was it found on other fungi commonly found in hazelnuts or chestnuts. However, the LAMP was not able to detect the strains BS3 (*A. flavus*), AFLX1 (*A. flavus*), AFLX2 (*A. flavus*), AFSP2 (*A. flavus*) and AFSP3 (*A. flavus*) which produced AFs in vitro, while by using the LAMP assay a positive amplification was obtained using the DNA of two *A. flavus* AFCAL7 and AFLX7 which were not able to produce aflatoxins in vitro.

The time to positive (Tp) ranged from 15 min 30 s to 37 min 17 s whilst the melting temperature ranged from 83.83 to 92.3.67 °C (Table 1 and Table 2). The sensitivity was tested against 10-fold serial dilutions of the DNA of the strains FS7 (*A. flavus*) and AFLX6 (*A. parasiticus*), showing a limit of detection of 100-999 pg/µL of DNA for both species (Table 4, Figure S6) with a Tp of the aflatoxigenic strains obtained in the three independent assays in a repetitive and reproducible way.

### 2.4. qPCR Validation

The primers and TaqMan^®^ probe sequences, which targeted 74 bp (product size calculated according to the *A. parasiticus* sequence of the aflatoxigenic biosynthetic cluster, GenBank accession number: AY371490) are listed in Table 3 and Figure S2**.** The primers aligned with 100% similarity against the sequenced product of the PCR performed with the AfF and AfR primers and the DNA of FP8, BV10 and FV5 and produced an expected amplicon of 74 bp in the three case. Regarding the localization in the *aflT* gene, the primers and probe were designed at the end of the last exon of the *aflT* gene (Figure S3). BLASTn analysis of the primers forward, reverse and the probe, showed 100% similarity with *A. flavus* NRRL3357, *A. flavus* AF36, *A. flavus* AF13, *A. flavus* BN008, *A. flavus* AF70 and other *A. parasiticus* and *A. flavus* (GenBank accession numbers: XM002379911.1, AY510455, AY510451, AY510452, AY510453, AY371490, AF515601, respectively).

SYBR™ Green assay was initially used to check the specificity of the primers and the melting temperature of the qPCR product. The Ct of the positive results ranged from 11.58 to 41.14 whilst the melting temperature of the positive results was between 78.74 and 80.97 °C. Three replications of each reaction were carried out, with some of the technical replicates showing positive amplification and, many non-aflatoxigenic strains were amplified, such as some non-aflatoxigenic, i.e., *A. flavus* strain AFLX9 and AFLX10. Therefore, in order to increase the specificity of the qPCR, a TaqMan^®^ probe was designed. In total, the SYBR green assay produced 33 real positives (aflatoxigenic strains), 24 real negatives (non-aflatoxigenic strains), 27 false positives and 13 false negatives. The sensitivity of the SYBR Green tested through a standard curve with 10-fold serial dilutions of the DNA from the *A. flavus* FS7, showed a limit of detection of 169 fg/µL of DNA (with only two out of three replicates detected at this dilution). The standard curve equation was y = −1.1313x + 25.624; with R^2^ = 0.967 using the ln of the DNA concentration, as shown in the Figure S7. The specificity assay for the qPCR with TaqMan^®^ probe showed 42 real positives (aflatoxigenic strains) including 3 out of 7 reference strains amplified (*A*. *subolivaceus* CBS 501.65, *A. thomii* CBS 120.51 and *A. minisclerotigenes* CBS 117635), whilst *A arachidicola* CBS 117610, *A. parvisclerotigenus* CBS 121.62, *A. oryzae* var. *oryzae* CBS 466.31 and *A. flavus* var. *columnaris* CBS 485.65 reference strains were not detected with this assay. In total, 8 false positives, 5 false negatives and 42 real negatives were detected. The Ct with the TaqMan^®^ probe ranged from 15.6 to 40.37. No amplification was obtained with the DNA of other fungi commonly found in hazelnuts (Table S2). However, the TaqMan^®^ qPCR was not able to detect the aflatoxigenic strains AFLX1, AFLX2 and AFSP2, whilst it gave positive amplification using the DNA of the *A. flavus* FP8, BV10, BS5, NP1, NP2, NP3, AFCAL7 and AFLX7 which were unable to produce aflatoxins in vitro.

Using the same DNA of the panel of inclusion-exclusion adjusted between 1 and 50 ng/µL, previously used for the LAMP assay, the Ct ranged from 19.80 to 30.10 (Table 1 and Table 2 and Table S1). The sensitivity of this assay was tested with the serial dilutions of the DNA of *A. flavus* FS7 and *A. parasiticus* AFLX6, starting with 16.9 ng/µL and 1.83 ng/µL, respectively. The qPCR with TaqMan^®^ probe showed an LOD of 16.9 fg/µL (only two replicated out of three) and 183 fg/µL (two replicates out of three).

The standard curve for *A. flavus* FS7 was y = −1.4471x + 24.844, R² = 0.9937, slope of 3.5394 and efficiency of 91.66%, whilst the standard curve using the ln of the DNA concentration of AFLX6 was y = −1.49x + 24.124, R^2^ of 0.987 with a slope of 3.42, and an efficiency of 96.06% (Figure S7).

### 2.5. In Vivo Validation

The sensitivity of both techniques (LAMP and qPCR with TaqMan^®^ probe) was evaluated using artificially inoculated hazelnuts in order to test the reliability of these techniques on hazelnuts as food matrix. Three different strains, previously characterized by Prencipe et al. [53] corresponding with one non-aflatoxigenic *A. flavus* (AFSP1), one aflatoxigenic *A. flavus* (AFSP4) and one aflatoxigenic *A. parasiticus* (AFLX6) were used to inoculate hazelnuts at three different concentrations. No amplification was shown in any dilution with any type of DNA extraction with the non-aflatoxigenic *A. flavus* isolate AFSP1.

Regarding the LAMP results, the type of DNA extraction (crude alkaline extraction or commercial kit) influenced the number of positive replicates at each concentration. With the crude DNA extraction, 3/9, 1/9 and 3/9 hazelnuts inoculated with the aflatoxigenic strain AFSP4 were detected at the concentrations of 3.03 × 10^5^, 3.03 × 10^6^, and 3.03 × 10^7^ spores/mL, respectively. The Tp ranged from 32 min 09 s to 58 min 05 s and the melting temperature from 88.76 to 89.21 °C. The approximate number of cells in the crude extract is reported in Table 5 and was calculated according to the standard curve of the TaqMan^®^ qPCR. The number of cells calculated with the TaqMan^®^ qPCR in each mL of crude extraction corresponded to 1.7 × 10^5^ cells, 1.1 × 10^4^ cells and 6.3 × 10^5^ cells, respectively. Instead, hazelnuts artificially inoculated with the strain AFLX6 were only detected in one and two out of nine replicates with an inoculum of 1.79 × 10^5^ and 1.79 × 10^6^ spores/mL, respectively. The number of cells calculated with the TaqMan^®^ qPCR was 5.6 × 10^5^ and 8.4 × 10^4^, respectively. Hazelnuts inoculated with 1.79 × 10^7^ spores/mL were not detected with the LAMP assay. The Tp ranged from 38 min 06 s to 52 min 46 s and the melting temperature from 88.17 to 89.23 °C.

In all cases, the cytochrome oxidase I (COX) LAMP assay was used as internal control and produced positive results in all the replicates with Tp ranging from 4 min 29 s to 15 min 40 s and melting temperature from 84.92 to 85.36 °C.

The number of positive LAMP results increased significantly when the DNA of the hazelnuts was extracted using a commercial kit. This commercial DNA extraction kit was also used to check the sensitivity of the qPCR with TaqMan^®^ assay in vivo. Regarding the hazelnuts inoculated with the AFSP4 strain, nine out of nine replicates were detected with the LAMP assay. In this case, the Tp ranged from 28 min 16 s to 50 min 16 s and the melting temperature ranged from 87.89 to 88.78 °C. The TaqMan^®^ qPCR produced repetitive results for all the concentrations tested with Ct ranging from 25.65 to 33.98 for the hazelnuts inoculated with 3.03 × 10^5^ spores/mL; 30.63 to 32.49 for the hazelnuts inoculated with 3.03 × 10^6^ spores/mL and 24.49 to 28.66 for the hazelnuts inoculated with 3.03 × 10^7^ spores/mL. The Ct was afterwards used to calculate the approximate number of cells in each µL of the DNA extraction by means of a standard curve. The lowest number of cells detected was 53 spores/mL (samples inoculated with 3.03 × 10^6^ spores/mL) and the maximum 6.2 × 10^4^ spores/mL for samples inoculated with 3.03 × 10^7^ spores/mL.

Regarding the hazelnuts inoculated with the strain AFLX6 and extracted with a commercial kit, they were detected in most replicates, 6/9 at the concentration of 1.79 × 10^5^ spores/mL, 8/9 at the concentration of 1.79 × 10^6^ spores/mL and 9/9 at the concentration of 1.79 × 10^7^ spores/mL, with Tp ranging from 30 min 24 s to 45 min 42 s and melting temperature ranging from 88.03 and 88.78 °C. The TaqMan^®^ qPCR produced repetitive results in all the concentrations of this strain tested. The Ct ranged from 26.90 to 39.89 for the hazelnuts inoculated with 1.79 × 10^5^ spores/mL, 29.91 to 37.10 for the hazelnuts inoculated with 1.79 × 10^6^ spores/mL, 25.63 to 31.11 for the hazelnuts inoculated with 1.79 × 10^7^ spores/mL. The lowest number of cells detected was 60 spores/mL and the maximum 1.9 × 10^5^ spores/mL for samples inoculated with 1.79 × 10^7^ spores/mL.

### 2.6. Naturally Contaminated Hazelnuts

The presence of *Aspergillus* spp. in the hazelnuts was checked by plating serial dilution into Aspergillus flavus and parasiticus agar (AFPA) medium. The presence of orange-to yellow colonies in the dilution 1 × 10^5^ confirmed the natural contamination of the lot.

Nineteen out of 50 hazelnuts extracted with a commercial kit showed positive amplification using the qPCR with the TaqMan^®^ probe. The Ct ranged from 33.79 to 39.99 which corresponded with 0.0003 to 0.024 ng of DNA and between 8 spores/µL and 612 spores/µL (Table 6). Though, no amplification was obtained with the LAMP assay with any type of DNA extraction, due to a lower sensibility of this technique compared with the qPCR with TaqMan^®^ probe.

### 2.7. Comparison between PCR, TaqMan^®^ qPCR, LAMP Assay and Aflatoxin Production

A positive correlation was found between the presence of *aflT*, detected by end-point PCR, with the production of AFs. Out of 97 strains tested in this study, from hazelnuts, chestnuts and CBS collection, only six strains did not present the band, but at least they produced one of the AFs (usually at least AFB1). These six isolates were all *A. flavus* (BS6, BS9, AFLX1, AFLX2, AFSP2 and AFSP3). These results suggested that in most of the strains, this region of the *aflT* was essential for the production of AFs, and it was exploited in order to design the LAMP assay and the qPCR with TaqMan^®^. However, there were also eight strains that presented the band but did not produce any AFs in vitro all identified as *A. flavus.* The diagnostic specificity and sensitivity were calculated for this assay, considering real positives—aflatoxin producers of at least one of the four aflatoxins investigated— as well as real negatives—non-aflatoxin producers in vitro. The PCR showed +LR of 4.85 and -LR of 0.16 meaning that approximate 1 in 1.2 positive results, are aflatoxigenic strains, whilst, 1 in 1.2 negative results are non-aflatoxigenic strains. The diagnostic sensitivity was 87.2% and the diagnostic specificity was 82.0% (Table 7).

A comparison between the number of real positives, real negatives, false positives and false negatives was carried out also for the LAMP and the qPCR (Table 7). With DNA from pure cultures of 97 strains (Table 1 and Table 2 and Table S1), the LAMP assay identified 42 real positives, 48 real negatives, 5 false negatives (the LAMP did not produce amplification, but the strain produced at least one aflatoxin in vitro) and 2 false positives (the LAMP was positive, but the strains did not produce any aflatoxin in vitro). The LAMP assay showed a diagnostic sensitivity of 89.4% and diagnostic specificity of 96.0%, with +LR of 22 and –LR 0.11 meaning that approximately 1 in 1.0 strains with a positive LAMP result are real aflatoxigenic strains, whilst approximately 1 in 1.1, with negative LAMP result are non-aflatoxigenic strains.

The qPCR assay with SYBR Green produced 33 real positives, 24 real negatives, 27 false positives and 13 false negatives that related to the +LR of 1.36 (1 in 1.8 positive results are aflatoxigenic strains) and –LR of 0.6 (1 in 1.5 negative results are non-aflatoxigenic strains). The diagnostic sensitivity of this assay was 71.7% and the specificity was 47.1%. All these values increased with the TaqMan^®^ qPCR (42 real positives, 42 real negatives, 8 false positives and 5 false negatives). The TaqMan^®^ qPCR assay showed diagnostics sensitivity of 89.4% and diagnostic specificity of 84.0% with +LR of 8.94 and –LR 0.12 meaning that approximately 1 in 1.1 strains with a positive TaqMan^®^ qPCR positive are real aflatoxigenic strains and 1 in 1.2 are non-aflatoxigenic strains when the TaqMan^®^ qPCR gives a negative result (Table 7).

The TaqMan^®^ qPCR assay was not able to detect *A. parvisclerotigenus* and *A. arachidicola*, but the LAMP assay showed a quick amplification using the DNA of these two strains.

Using the data from the artificially inoculated hazelnuts, and considering each biological replicate as an individual sample (81 samples in total), the LAMP using the crude extraction method has a diagnostic sensitivity of 18.5% and diagnostic specificity of 100%, with +LR of 999999 and –LR 0.81, meaning that approximately 1 in 1.0 strains with a positive LAMP result are real aflatoxigenic strains, whilst approximately 1 in 2.6 with negative LAMP result are real non-aflatoxigenic strains. Regarding the results of the LAMP with the DNA of the hazelnuts extracted using a commercial kit, the diagnostic sensitivity and specificity were 92.6% and 100%, respectively, with +LR of 999999 and –LR 0.07 meaning that approximately 1 in 1.0 strains with a positive LAMP result are real aflatoxigenic strains, whilst approximately 1 in 1.1 with negative LAMP results are true non-aflatoxigenic strains. Instead, the TaqMan^®^ qPCR assay showed diagnostics sensitivity and specificity of 1, with +LR of 999999 and –LR 0.00 meaning that approximately 1 in 1.0 strains with a positive TaqMan^®^ qPCR are real aflatoxigenic and 1 out of 1 with a negative TaqMan^®^ qPCR results are non-aflatoxigenic strains.

In connection with the deletion of the aflatoxigenic cluster, three strains (*A. flavus* FP8, BV10 and BS5) showed a positive amplification with the end-point PCR, and the qPCR assay, but no positive result was found with the LAMP.

The *A. flavus* strains AFLX1, AFLX2 and AFSP2 reported as aflatoxigenic, were not detected by any technique (LAMP or qPCR), but *A. flavus* AFSP3 was only detected by the TaqMan^®^ qPCR assay and not using the LAMP assay. None of these strains amplified with the end-point PCR primers.

## 3. Discussion

Presence of aflatoxigenic molds in food and feed is a critical threat to consumer safety and to the industry. The most effective management actions focus on the reduction in aflatoxin contamination in food, while maintaining the food sanitary standards [54].

Riba et al. [55], reported that 51.8% of the *Aspergillus* section *Flavi* strains found in roasted hazelnuts were aflatoxigenic, confirmed in our data which showed that 48% of the hazelnut strains were able to produce AFB1 in vitro. In addition, the AFB2 was also produced in most of the strains that produced the AFB1.

Among *Aspergillus* section *Flavi*, *A. flavus* has been reported more predominant than *A. parasiticus* in hazelnuts [14]. Traditionally, the *Aspergillus* species identification in food matrices is based on plating methods on selective media that are time-consuming and labor-intensive [56]. *A. oryzae*, a non-aflatoxigenic species, is the most phylogenetically related species to *A. flavus*, as *A. sojae* and *A. nomius* to *A. parasiticus* and *A. toxicarius*. For example, *A. parasiticus* and *A. sojae* can be identified accordingly to the color of colonies, the conidial diameter or using a bleomycin media [57]. However, these differences are not always clear due to the changes in the morphological features caused by the environment, and an accurate identification needs a lot of experience to differentiate between the species. In addition, it has been reported that *A. flavus* and *A. parasiticus* after long culturing may change their morphological features to appear like *A. oryzae* and *A. sojae* and *A. nomius*, respectively [58]. Currently, sequencing of barcoding genes such as ITS (internal transcribed spacer), beta-tubulin gene, RNA polymerase II gene (RPB2) or calmodulin gene [4,53,59,60] can resolve almost all the species of *Aspergillus* section *Flavi*, however, they cannot distinguish between *A. flavus* and the phylogenetically related and non-aflatoxigenic species *A. oryzae,* or between *A. parasiticus* and *A. sojae* [4]. Although, in the food-producing industry, it is more critical to determinate the aflatoxigenic potential of the strain than identify the species and this requires a combination of multiple procedures such as categorizing the species in function of the micro and macro morphological features, barcoding and the quantification of the secondary metabolites [29,53,59].

Considering the time necessary for culturing and the challenges associated with the morphological identification, other techniques have been used to determine the presence of aflatoxigenic fungi in foodstuffs. PCR-based assays are widely useful in laboratories due to their specificity, sensitivity and accurate detection properties. They are essential to identify non-predominant species without the pre-enrichment step of growing in a selective medium. However, due to the high degree of DNA relatedness between *A. flavus* and *A. oryzae* and *A. sojae, A. nomius* and *A. parasiticus* [5,61], the design of PCR-based methods for the identification of different *Aspergillus* spp. can be a challenge. Although barcoding genes (calmodulin or beta tubulin) are widely used for phylogenetic purposes, they do not help in distinguishing between aflatoxigenic and non-aflatoxigenic strains. Other genes involved in the aflatoxin biosynthetic pathway can be exploited, such as the *omt-A,* or the *aflR*, that have been used by some authors to design end-point PCR or TaqMan^®^ qPCR to detect and quantify aflatoxin-producing *Aspergillus* spp. [23,33,36,62]. LAMP assays have been designed in other genes such as alpha amylase 1 (amy1) to detect *A. flavus* or ATP citrate lyase subunit 1 (acl1) to detect *A. nomius* and *A. parasiticus* [44] in Brazil nuts, peanuts and green coffee beans, similarly to the qPCR assays. The aflatoxigenic biosynthetic gene cluster has been already exploited to design LAMP assay methods on genes such as *aflP, aflD* or *nor1* to distinguish between aflatoxigenic and non-aflatoxigenic fungi in different food matrices [46,48,50].

Within the aflatoxin biosynthetic gene cluster, some studies reported the positive relationship between the lack of some regions with the inability to produce aflatoxins. The region *norB* – *cypA* showed to present some deletions in the non-aflatoxigenic isolates [9], and the total lack of the aflatoxin cluster has been shown in some strains. Chang et al. [9] reported eight patterns of the cluster according to the lack of some regions. Four types have been described according to the deletions identified within *norB*–*cypA* regions depending on the amino acids affected and the intergenic region. While four patterns have been characterized by presenting a deletion larger than 45 where the entire aflatoxin gene cluster was lost, even affecting downstream sugar utilization gene cluster (*nadA-hexA-glcA-sugR*).

Designing primers to obtain specific and sensitive diagnostic tests can be a hard task, and many challenges were faced in this study in order to obtain reliable tests which exploited the differences in the aflatoxigenic biosynthetic cluster between aflatoxigenic and non-aflatoxigenic strains. In this case, we profited from a region of the *aflT* gene [63], whose deletion was positively correlated with the absence of aflatoxin production in chestnuts and hazelnuts strains. This region allowed us to obtain three types of diagnostic assays that could be applied in different scenarios, as a quick test in the laboratory (end-point PCR), as a quick and user-friendly on-site test (LAMP) and for the quantification of the aflatoxigenic strains and confirmation of samples with low amount of inoculum (qPCR with TaqMan^®^ probe). The latter two methodologies were validated according to European Mediterranean Plant Protection Organization (EPPO) PM 7/98 standards in terms of specificity, sensitivity, repeatability and reproducibility with DNA obtained from pure culture and from hazelnuts. For the LAMP assay, in order to obtain specific primers, instead of using software, primers were manually designed following the guidelines in the Eiken Genome Site (http://loopamp.eiken.co.jp/e/lamp/primer.html) in terms of distance between primer regions, GC content and secondary structure. Out of 97 strains used in this study to confirm the specificity of all the techniques, considering also the CBS reference stains, only six did not present the band and did produce AFs. However, three out of these strains gave a positive result with the TaqMan^®^ qPCR but not with the LAMP. Instead, nine strains presented the band and did not produce any aflatoxin.

Quantification of the mycotoxins in the different matrixes usually requires analytical techniques to quantify these secondary metabolites. Mycotoxins are usually produced at low concentrations, in consequence, liquid chromatography associated with mass spectrometry or fluorescence detection has become a unique method for the quantification of mycotoxins due to the high sensitivity and reliability [64]. Differently from previously published assays [50], we confirmed the aflatoxigenic potential of all the strains used in this study with a robust and sensitive analytical method which showed an LOD of 0.1, 0.2, 0.4, and 0.2 ppb and an LOQ of 0.5, 0.7, 1.2, and 0.8 ppb for AFB1, AFB2, AFG1, and AFG2, respectively. The matrix effect of YES broth was also taken into consideration to calculate the LOQ.

Nonetheless, it is possible that AFs production was below the LOQ of the analytical method (although the method was sensitive) in the nine strains that present the PCR band but no AFs production. However, the most likely explanation, that confirms what is reported in literature, is that the in vitro assay could underrate the aflatoxigenic potential of strains. This idea can explain that some of the strains, which did not produce AFs in vitro, resulted in a positive LAMP or qPCR results as it is likely that they produce aflatoxins in vivo [53,65]. As the objective of this study was the design of techniques that can detect or quantify aflatoxigenic fungi, but not to quantify the level of AFs, HPLC analyses were used as a standard method to validate the molecular techniques. Despite, the end-point PCR assays showed a high specificity of 82.0% with an LOD of 110 fg/µL of DNA. In terms of sensitivity, the LAMP assay designed here, showed a limit of detection of 100–999 pg/µL of DNA, like the limit of detection previously described in other assays [66,67]. However, Niessen et al. [50] also developed a LAMP assay that focused on the detection of aflatoxigenic strains of *Aspergillus* section *Flavi*, with primers designed on the *nor1* gene, with a lower detection limit of 9.03 pg of genomic DNA per reaction and based on the detection of the positive results by a colorimetric change or by a fluorescence dye. Although the LOD of our LAMP was higher than that of other assays, we obtained a more sensitive diagnostic test with a TaqMan^®^ qPCR that detected 169 fg/µL of genomic DNA of *A. flavus* and 0.0003 ng/µL of DNA of hazelnuts naturally contaminated by aflatoxigenic strains, corresponding with eight spores/µL. The diagnostic sensitivity was 89.4% both for LAMP and qPCR.

Both techniques, TaqMan^®^ qPCR and LAMP, showed high specificity using DNA from pure culture samples, with no amplification of any other fungi commonly found in hazelnuts. In both cases, the diagnostic specificity and sensitivity were higher than in other studies (96% for the LAMP and 84% for the TaqMan^®^ qPCR), overcoming other assays such as the LAMP designed by Douksouna et al. [48], to detect aflatoxigenic molds in rice, with a specificity of 71.5% using pure DNA. In addition, both tests were validated in terms of repeatability and reproducibility following the EPPO PM7/98 validation standards. Melting temperatures of the SYBR (ranging between 79 and 80 °C) and the LAMP (ranging from 88 to 89 °C) assays were also included as key parameters for the interpretation of the results.

Although both LAMP and qPCR were validated to detect the most common *Aspergillus* species that are hazelnut contaminants (*A. flavus* and *A. parasiticus*), we have seen positive amplification with the DNA of other species such as *A. toxicarius*, *A. subolivaceus, A. thomii, A. arachidicola, A. minisclerotigens* and *A. parvisclerotigenus.* Currently, *Aspergillus* section *Flavi*, includes 15 species, 5 of them recently described [4], with four well supported lineages. The two main lineages are centered in *A. flavus* (including A. *minisclerotigenes, A. subolivaceus, A.* var. *columnaris, A. thomii* and *A. oryzae*) and centered in *A. parasiticus* (including *A. toxicarius, A. arachidicola* and *A. sojae*) [4]. Although in vivo validation of these assays were only performed with *A. flavus* and *A. parasiticus* spores, the LAMP and TaqMan^®^ qPCR could be potentially used to detect the DNA of other species of the *Aspergillus* section *Flavi.*

It is important to highlight an important caveat in the specificity, as some of the LAMP primers showed 100% similarity in silico with non-aflatoxigenic strains such as. *A. sojae* or *A. pseudotamarii.* It was confirmed that the LAMP or TaqMan^®^ qPCR did not amplify *A. sojae* (isolate BS8), however, we only confirmed that both qPCR and LAMP primers did not amplify the DNA of *A. tamarii,* which shares 90% similarity with *A. pseudotamarii* and belongs to the same lineage [4]. In addition, LAMP assays are less sensitive to inhibitors present in the matrix than other molecular techniques allowing the use of crude extraction based on the alkaline disruption of the membrane of the cells to extract the DNA [68]. Previous LAMP assays did not test the sensitivity of the technique with real samples, artificially or naturally infected samples [48] However, it is essential to evaluate the potential application of the assays in real samples. No inhibitions were shown using hazelnuts samples with any of the two assays. During the sensitivity assay using artificially inoculated hazelnuts, repetitive results were obtained using the commercial DNA extraction, within the replicates of the aflatoxigenic *A. flavus* and the *A. parasiticus* and no amplification was obtained for the non-aflatoxigenic *A. flavus* confirming the specificity of both techniques in vivo.

However, not all the LAMP tests gave positive amplification with the crude DNA extracts. This type of crude extraction dilutes the samples over 50,000 times, and it has been a suitable type of extraction for another type of matrix such as plant material, seeds or fruit [52,66,69]. However, in this case it did not identify samples with a low concentration of inoculum.

In some situations, the EPPO (European Mediterranean Plant Protection Organization) protocols suggest the combination of methods rather than individual tests [70]. The low admitted levels of aflatoxins in food in the European Union [71] require the measurement of mycotoxin concentration in the different foodstuff matrixes by HPLC as it might be possible to find lots with concentrations that exceed legal limits [72]. The risk to human health of foodstuffs contaminated with aflatoxins makes it essential to detect aflatoxigenic fungi, and therefore, to evaluate the uncertainly of each diagnostic test in terms of false positives and false negatives. In some situations, such as routine diagnosis, the speed of the analysis could be the most important parameter, so that a single technique, such as the LAMP, could be used as a screening method that will speed up further decisions. Then, despite the negative results of the LAMP on hazelnuts using a crude extraction method and a sensitivity of 100–999 pg/µL of DNA, the assay on portable devices such as the Genie II instrument could be used as an initial screening method per batch, avoiding misreading of color changes. We suggest that negative results should then be confirmed with the TaqMan^®^ qPCR assay (with higher sensitivity of 160 fg/µL of DNA or 8 spores/µL using DNA from hazelnuts) or the LAMP using a more purified extraction method, such as a commercial DNA extraction, which is more sensitive. In addition, the use of the end-point PCR primers and the LAMP assay could be used on the DNA of pure cultures of the *Aspergillus* section *Flavi* spp. to confirm the aflatoxigenic potential in some hours or in less than 40 min, respectively. However, HPLC methods, as the one used here, remain a standard method for quantifying the level of aflatoxins.

## 4. Conclusions

In summary, the direct analysis of hazelnuts, instead of DNA extraction from pure cultures obtained 7 to 14 days after plating, will significantly reduce the time between sampling and result obtainment. This will help greatly in the decision-making behind keeping or discarding a lot of nuts in the food industry, due to possible aflatoxin contamination. The methods developed here, the LAMP assay or the TaqMan^®^ qPCR assay, identified with high confidence the aflatoxigenic *A. flavus,* and *A. parasiticus* in both pure DNA and hazelnuts. After the validation following an international EPPO standard (PM7/98), both techniques may be used as complementary to each other to identify the presence of aflatoxigenic strains in food matrices. Positive samples could be confirmed by HPLC methods in order to precisely quantify the AFs levels in the food matrix.

## 5. Materials and Methods

### 5.1. Isolate Collection and DNA Extraction

Ninety *Aspergillus* spp. listed in Table 1 and Table 2 were used for the validation of both LAMP and qPCR assays. Fifty strains (Table 1) were isolated from hazelnut leaves (24), bracts (16) and nuts (8) and forty (Table 2) from different steps of the chestnut production chain [57]. Moreover, seven reference strains of *Aspergillus* spp. purchased from the Westerdijk Fungal Biodiversity Institute were included to validate the results of the LAMP and the qPCR assays (Table S1). These 7 species were selected as they were part of the two main lineages of *Aspergillus* section *Flavi*, the one centred in *A. flavus* and the other centered in *A. parasiticus* [4]. Single-spore cultures of isolates were grown in YES agar as described by Frisvad [73] for 7 days at room temperature according to the protocol described by Visagie et al. [74]. The mycelium of each isolate was collected from the agar plate and stored at −20 °C. Total genomic DNA was obtained using the E.Z.N.A. Fungal DNA mini kit (OMEGA Bio-Tek, Norcross, GA, USA), according to the manufacturer’s instructions and stored at −20 °C. DNA concentrations were measured using NanoDrop 2.0 (ThermoFisher, Wilmington, DE, USA) and adjusted to 1–50 ng/µL. The same genomic DNA was used to check the specificity of both LAMP and qPCR assays. Molecular identifications of *Aspergillus* section *Flavi* obtained from hazelnut trees were performed using the primers cmd5 (5′-CCGAGTACAAGGARGCCTTC-3′) and cmd6 (5′-CCGATRGAGGTCATRACGTGG-3′) [75] to amplify the calmodulin gene. The PCR reactions were performed in 20 µL with approximately 50 ng of genomic DNA, 2.5 mM deoxynucleotide triphosphates, 2.5 mM MgCl2, 0.5 µM of each primer, QIAGEN reaction buffer diluted 1:10 and 1U of Taq DNA polymerase (QIAGEN, Chatsworth, CA, USA) in a T-100 thermal cycler (Bio-Rad Japan, Tokyo, Japan). The protocol for the calmodulin was set as described by Prencipe et al. [53]. The PCR products were checked by gel electrophoresis at 1% agarose. PCR products were sequenced using the external service of GATC services (Costanza, Germany) in both directions. Contigs of forward and reverse sequences were carried out using the DNA Baser program (Heracle BioSoft SRL, Pitești, Romania).

Alignment of the sequences was performed in CLUSTALW (multiple sequence alignment) using MEGA 6 program [76] with default parameters. The calmodulin sequences of some the isolates listed in the Table S3, previously identified by Frisvad et al. [4] were included in the alignment, as well as the sequence of some chestnut isolates obtained from GenBank (isolates AFSP6, AFCAL9 and AFSP8). The sequences were then edited manually and trimmed obtaining a data set of 461 bp.

The best-fit model (K80 +G) was determined using the tool Model Selection in Topali v2.5 program [77] for maximum likelihood that was carried out using the same software with 100 bootstrap replicates to evaluate the stability. Cladogram tree topologies were adjusted using FigTree v1.4.3 and represented in the Figure S1.

### 5.2. In vitro Aflatoxin Production

The aflatoxigenic potential of the strains listed in Table 1 and Table 2 and the 7 reference strains (Table S1) was evaluated in an in vitro assay with HPLC coupled fluorescence detection. The protocol used to induce aflatoxin production was based on the in vitro assay described by Prencipe et al. [53]. The strains were grown on YES agar [71] for 1 week at 30 °C in the dark. Three agar plugs (1 cm diameter) were used to inoculate 50 mL of YES broth [74], subsequently incubated for one week at 30 °C in the dark.

The culture broth was filtered through a Whatman filter paper, grade 1 (VWR International, Milan, Italy), to check the aflatoxin production. The broth (10 mL) was extracted with ethyl acetate (Sigma Aldrich, Steinheim, Germany) performing the liquid–liquid extraction twice. The organic extracts were mixed and evaporated to dryness in a rotatory evaporator. The residue was dissolved in 0.5 mL of methanol-water (50:50 *v/v*) for HPLC analysis. All the samples were diluted to remain in the linearity range. All the strains were grown in triplicate under the same conditions, and AFs were extracted twice.

Analyses were performed using an Agilent 1260 Series (Agilent Technologies, Santa Clara, CA, USA) equipped with a fluorescent detector and an electrochemical cell (KOBRA^®^ CELL, R-biopharm, Darmstadt, Germany) for the post-column derivatization. Chromatographic separation was performed on a Pursuit XRs Ultra C18 (100 mm × 2.0 mm, 2.8 µm, Varian) column in isocratic mode (80:20) using H_2_O:CH_3_OH:CH_3_CN (50:40:10 *v/v/v*) acidified with 0.035% HNO_3_ and 119 mg/L KBr in H_2_O at flow rate of 1 mL/min for 15 min. Quantification of AFs was performed by external calibration method using standard curves of the four aflatoxins (AFG1, AFG2, AFB1, AFB2, Sigma Aldrich, crystallized form) at the concentration range of 0.1 to 25 ppb. The quantity of AFs reported is the average of six replicates (three biological replicates and two technical replicates per biological one).

### 5.3. HPLC-FLD Method Validation

The developed analytical method was evaluated for recovery, limit of detection (LOD), limit of quantification (LOQ) and matrix effect (ME) for the four aflatoxins in accordance with Matuszewski et al. [78] and Kos et al. [79]. Analytical standards of AFB1, AFB2, AFG1, and AFG2 (Sigma-Aldrich) were used to prepare two sets of samples: (i) standard solution dissolved in mobile phase in a range of concentrations from 0.1 up to 25 ppb; (ii) spiked matrix: the extraction was performed from YES broth media, afterwards, the four aflatoxin standards were added in the range from 0.1 up to 25 ppb. Signal-to-noise method was used to determine limits of detection (LOD) and quantification (LOQ) for the mobile phase and the matrix. S/N ratio 3:1 was used for the determination of LOD, while 10:1 for LOQ.

### 5.4. Confirmation of Deletion in the Aflatoxin Gene Cluster. PCR LOD

Two manually designed primers (AfF and AfR) were used to check the presence or absence of the region in the aflatoxin biosynthetic gene cluster where the LAMP and qPCR primers were later designed. The primers were designed flanking the region in which the LAMP primers were designed amplifying a region of approximately 308 bp. Possible secondary structures of the primers were also tested as explained above.

A PCR reaction using AfF and AfR of each strain used in this study (Table 1 and Table 2), was performed in 20 µL with approximately 50 ng of genomic DNA, 2.5 mM deoxynucleotide triphosphates, 2.5 mM MgCl_2_, 0.5 µM of each primer, Qiagen reaction buffer diluted 1:10 and 1U of Taq DNA polymerase (Qiagen, Düsseldorf, Germany) in a T-100 thermal cycler (Bio-Rad Japan, Tokyo, Japan). The protocol included an initial denaturation step at 95 °C for 3 min and 34 cycles, including 95 °C for 30 s, 59.5 °C for 30 s and 72 °C for 1 min, with a final extension at 72 °C for 7 min. The PCR products were checked by gel electrophoresis at 2% agarose (VWR International, Milan, Italy). Limit of detection of the technique was calculated using 10-fold serial dilutions of the *A. flavus* FV3 (initial concentration 110 ng/µL).

### 5.5. LAMP and TaqMan^®^ Assay Primers Design

The primers for LAMP and qPCR were designed on the biosynthetic aflatoxin gene cluster, specifically aligning to the *aflT* gene (transmembrane protein; at the end of the last exon and the beginning of the last intron), in order to detect and quantify the DNA of the aflatoxigenic *Aspergillus* section *Flavi* strains, with special interest in *A. flavus* and *A. parasiticus*. Due to the difficulty to find specific primers with any software, the six LAMP primers were manually designed: two external primers (F3 and B3), two loop primers (F-loop and B-loop) and two internal primers (BIP and FIP) following the instructions described on-line [80,81]; two primers and a TaqMan^®^ probe were designed for quantitative PCR in the same region (Figures S2 and S3). A multilocus alignment of the complete biosynthetic aflatoxin gene cluster was performed using the sequences of *A. flavus* (GenBank accession number AY510453.1, AY510455.1, AY510452.1), *A. oryzae* (GenBank accession number AB196490.1), *A. parasiticus* (GenBank accession number AY371490.1) and *A. nomius* (GenBank accession number AY540454.1) as shown in the Figure S2. The alignment was carried out using CLUSTALW (multiple sequence alignment) using MEGA 6 program [74] with default parameters. Possible secondary structures, hairpins, and self-annealing were checked for six LAMP primers, the TaqMan^®^ probe and the two qPCR primers using OligoCalc (http://biotools.nubic.northwestern.edu/OligoCalc.html). BLASTn analysis of all the primers and the TaqMan^®^ probe (National Center for Biotechnology Information, https://blast.ncbi.nlm.nih.gov/) was also carried out to check the specificity. In order to verify the LAMP and qPCR products, the products of the PCR amplicon obtained with DNA of *A. flavus* FP8, BV10 and BS3 and with the AfF and AfR primers were sequenced using services of Macrogen Europe (Amsterdam, The Netherlands). The primers of the LAMP, qPCR and the probe were then aligned against the three sequences. In order to understand the localization of the primers, they were aligned against the sequence (exons and introns) of the *aflT* from *A. flavus* (GenBank: AF515601.1). All primers were synthesized and HPLC purified by Eurofins (UK). Probe for the qPCR assay was synthesized by Metabion International AG (Planegg, Germany).

### 5.6. LAMP and qPCR Reactions

The LAMP reaction was carried out with 1 µL of DNA for the strains listed in Table 1 and Table 2, Tables S1 and S2 with concentration adjusted to around 1–50 µg/µL or 1 µL of crudely extracted DNA of artificially inoculated and real sample hazelnuts, as explained below, or 1 µL of the DNA extracted using E.Z.N.A. Plant DNA kit (Omega Bio-tek, Inc., Norcross, GA, USA) from inoculated and real sample hazelnuts.

The LAMP reaction was performed in 25 µL including 0.3 µmol/L of the external primers F3 and B3, 1.2 µmol/L of each loop primer (F-loop and B-loop) and 2.3 µmol/L of each internal primer (FIP and BIP) and 1× of the Isothermal Mastermix ISO-004^®^ which contains a ds-DNA binding fluorescent dye (FAM detection channel) (Optigene Ltd., Horsham, UK). The reaction was performed as described by Franco Ortega et al. [43] for 60 min at 65 °C, recording the melting temperature whilst cooling from 95 to 70 °C at 0.05 °C/s using a Genie II^®^ instrument (Optigene Ltd.) and a StepOne thermal cycler (Applied Biosystems, Loughborough, UK). Positive controls, which included two strains able to produce aflatoxins (the AFB1 and AFB2 producer strain FS7 and previously identified as *A. flavus* and the *A. parasiticus* strain AFLX6 fungal DNA) and two negative controls, one non-aflatoxigenic strain (*A. flavus* AFSP1) and one with water, were included in each run. The average time to positive (Tp) of three technical replicates was used to calculate the Tp of each sample. Cytochrome oxidase I (COX) LAMP reaction was also performed using the protocol described by [52] as an internal control to confirm the presence of hazelnut DNA after the crude DNA extraction.

The qPCR assay was initially performed using the two designed qPCR primers (AflF and AflR) with SYBR™ Green to determine the melting temperature of the amplicons, and afterwards with the same two primers (AflF and AflR) and the TaqMan^®^ Afl-probe designed for this study.

In the SYBR Green experiment, the reaction was carried out with 0.3 µmol/L of each primer, 1× of the Power SYBR™ Green Master Mix (Thermo Fisher) and 1 µL of the DNA of each isolate listed in Table 1 and Table 2, Tables S1 and S2. The reactions were performed using a StepOne instrument (Applied Biosystems) with the following conditions: initial denaturation at 95 °C for 4.5 min, 40 cycles of 15 s at 95 °C and 15 s at 60 °C. The melting temperature using SYBR™ Green was measured while heating from 60 to 95 °C at 0.03 °C/s. Each reaction was performed in triplicate and positive (*A. flavus* FS7 and *A. parasiticus* strain AFLX6 fungal DNA) and negative controls (non-aflatoxigenic AFSP1 and water) were included in each run.

The same DNA was used to perform the qPCR with a TaqMan^®^ probe, using a final concentration of 0.3 µmol/L of each primer (AflF and AflR), 0.1 µmol/L of the TaqMan^®^ probe, 1× of TaqMan^®^ Universal PCR MasterMix (Applied Biosystems) and 1 µL of the DNA of each isolate listed in Table 1 and Table 2, Tables S1 and S2 or 1 µL of the hazelnut DNA (inoculated and real samples) extracted by using the E.Z.N.A. Plant DNA kit (Omega Bio-tek, Inc.). This assay was not performed using the DNA from the crude extracted hazelnuts. The reactions were performed using a StepOne thermal cycler with the setup explained above. Positive controls (*A. flavus* FS7 and *A parasiticus* AFLX6 fungal DNA) and negative controls (the non -aflatoxigenic strain AFSP1 and water) were included in each run.

The average of three technical replicates of the qPCR assay with TaqMan^®^ probe was used to calculate the number of cells present in the DNA extraction from artificially and naturally infected hazelnuts. The Ct values generated by the TaqMan^®^ Real Time qPCR assay were compared against the standard curve to calculate the amount (ng) of DNA in 1 µl of each positive result. The standard curve, to quantify the aflatoxigenic *Aspergillus* present in hazelnuts, was built using the DNA of the aflatoxigenic strains: FS7 (concentrations ranging from 16.9 ng/µL to 16.9 fg/µL) and *A. parasiticus* isolate AFLX6 (concentrations ranging from 1.81 ng/µL to 18.3 fg/µL). The standard curve generated with FS7 was used to quantify hazelnuts inoculated with *A. flavus*, as the standard curve generated with *A. parasiticus* AFLX6 was used to quantify hazelnuts inoculated with the latter strain. To quantify naturally infected samples, the standard curve generated with the *A. flavus* FS7 was used, as *A. flavus* is the most common aflatoxigenic species found in hazelnuts [14]. In order to calculate the number of cells in the inoculated hazelnuts, the elution volume of the commercial DNA (30 µL) of the inoculated hazelnuts was also taken into consideration, as described in other in vivo tests [67].

In all cases, the approximate number of cells detected was calculated by dividing the DNA quantity by the weight of the genome of *Aspergillus flavus* strain NRRL3357 (0.0000397 ng).

### 5.7. LAMP and qPCR Validation

Validation of the LAMP assay and the TaqMan^®^ qPCR was performed according to the EPPO standard PM7/98 in terms of specificity, sensitivity, repeatability, and reproducibility. The specificity testing included all the *Aspergillus* spp. in Table 1, 2 and Table S1 and other common fungi found in hazelnuts and in chestnuts (Table S2). The sensitivity testing of the LAMP and qPCR assays was carried out using 10-fold serial dilution of DNA of two aflatoxigenic species (*A. flavus* strains FS7 and *A. parasiticus* strain AFLX6) and in the case of the LAMP, the amplification was also confirmed through an agarose gel. The sensitivity was also related to the in vitro aflatoxin production and later, confirmed on artificially inoculated hazelnuts with the isolates: AFSP1 (non-aflatoxigenic *A. flavus*), AFSP4 (aflatoxigenic *A. flavus*) and AFLX6 (aflatoxigenic *A. parasiticus*), at different concentrations. All the samples were tested in triplicate, and an average of the obtained results was reported (repeatability). Reproducibility was checked by repeating the experiment in different machines, in different days and by two members of the staff.

Analytical specificity and sensitivity of all the assays (PCR, LAMP and qPCR with SYBR Green and TaqMan^®^ probe), using DNA of 97 *Aspergillus* spp. were evaluated using the calculator tool Diagnostic Test Calculator [82]. Diagnostic sensitivity (DSe) or true-positive rate and analytical specificity (DSp) or true-negative rate, were calculated using the following formulas: Dse = ΣTP/(ΣTP + FN); Dpe = ΣTN/(ΣTN + FP); where TP (True Positive) represents the aflatoxigenic strains, that showed a positive amplification with LAMP or TaqMan^®^ qPCR, TN (True Negative) denotes the non-aflatoxigenic strains that gave a negative result using LAMP or TaqMan^®^ qPCR, FN (False Negatives) indicates samples which gave a negative result using the LAMP or TaqMan^®^ qPCR assay but able to produce at least one of the 4 AFs, and FP (False Positives) are the samples showing positive amplification of LAMP or TaqMan^®^ qPCR but did not produce any AFs. The likelihood positive ratio (LR+) and the likelihood negative ratio (LR−) were calculated according to the ratio between DSe and the FP-rate and the FP-rate and Dse independently for the LAMP and for the qPCR. In addition, these parameters were also calculated for the artificially inoculated hazelnuts, considering as separate assays the LAMP results obtained with crude and the commercial DNA extraction. In this case, each individual technical replicate was used for the calculation.

### 5.8. Validation Using Artificially Inoculated Hazelnut

A batch of Italian hazelnuts kindly provided by Ferrero S.p.A. (Alba, Italy) was used to perform the sensitivity test of both LAMP and qPCR techniques in order to check the lowest detectable level of the fungi.

The hazelnuts were previously washed in water with 1% Tween 80 (Sigma Aldrich, Gillingham, UK) for 5 min, treated with sodium hypochlorite (2%), rinsed with distilled water and then air-dried for 30 min.

At the same time, spore-suspensions of three different *Aspergillus* spp. used for the specificity test were prepared. The non-aflatoxigenic *A. flavus* strain AFSP1, the aflatoxigenic *A. flavus* strain AFSP4 and the aflatoxigenic *A. parasiticus* strain AFLX6, were previously grown in YES agar at 30 °C for 10 days, as previously described. Spores-suspension with water and Tween 1% was obtained from the YES-agar plates. The spore concentrations were adjusted by means of a hematocytometer, obtaining 3.09 × 10^7^ for AFSP1, 3.03 × 10^7^ for AFSP4 and 1.79 × 10^7^ for AFLX6. Hazelnuts were artificially inoculated under sterile conditions with 10-fold serial dilution of the three strains: the non-aflatoxigenic *A. flavus* AFSP1 was inoculated with concentrations 3.09 × 10^7^, 3.09 × 10^6^, 3.09 × 10^5^ spores/mL, *A. flavus* AFSP4 was inoculated with concentrations 3.03 × 10^7^, 3.03 × 10^6^, 3.03 × 10^5^ spores/mL and *A. parasiticus* AFLX6 with concentrations 1.79 × 10^7^, 1.79 × 10^6^, 1.79 × 10^5^ spores/mL in independent assays. The inoculation was performed by immersion of 20 hazelnuts in 50 mL of spore suspension for 30 min in agitation (100 rpm) at room temperature. Hazelnuts were then air-dried overnight.

The DNA was extracted from six hazelnuts (for each inoculated concentration) with two different approaches. Three hazelnuts were used to perform a crude DNA extraction based on an alkaline extraction modified from the protocol described by Chomczynski and Rymaszewski [83] and subsequently used to perform a LAMP assay. The crude extraction buffer was composed by 1 mL of pH 13 PEG buffer (50 g/L PEG average Mm of 4600; 20 mmol/L KOH; pH 13.5) as described in Franco Ortega et al. [43] and were placed in a 5 mL tubes and homogenized by vigorous manual shaking for one minute. The solutions were then diluted 1:50 using 10 µL of crude extract in 490 µL of water. The other three hazelnuts of each concentration were ground with liquid nitrogen, extracted using E.Z.N.A. Plant DNA kit (Omega Bio-Tek) according to manufacturer’s instructions and eluted in 30 µL of water.

LAMP assay was performed using 1 µL of both DNA extraction methods in three different assays (technical replicates), whilst the TaqMan^®^ qPCR assay was performed in triplicate using 1 µL of the DNA obtained with a commercial extraction kit. The results of both types of extraction in the LAMP testing were then compared.

### 5.9. Validation Using Real Samples

Fifty hazelnuts were used to check the reliability of both assays, LAMP and qPCR, using naturally contaminated samples. The hazelnut shells were removed and the hazelnuts were extracted with both types of DNA extraction (crude DNA extraction and using the E.Z.N.A. Plant DNA kit eluted in a final volume of 30 µL) as described before. LAMP assay was performed using 1 µL of both DNA extracts in three independent replicates, whilst the qPCR assay was performed in triplicate using only 1 µL of the extract obtained with the commercial DNA extraction kit. The approximate number of cells on the crude DNA extract was calculated considering the average Ct obtained with 1 µL of the hazelnuts extracted with a commercial kit and using the standard curve of the strain FS7. The elution volume (30 µL) was also considered to calculate the approximate number of cells per hazelnut.

Fifteen hazelnuts from the same batch were used to check the presence of *Aspergillus* spp. using a traditional approach. Hazelnuts were agitated in 100 mL sterile water with 1% Tween solution for 2 h. The suspension was serially diluted (1:10, 1:10^2^, 1:10^3^, 1:10^4^ and 1:10^5^) and plated onto AFPA medium [84] in triplicate. Sampling of the *Aspergillus* spp. and the incidence was calculated after two days of incubation at 30 °C in the dark. The identification was performed visually according to the presence of orange-yellow pigmentation on the reverse side of the colonies [53].

## Figures and Tables

**Table 1:** Strains from hazelnuts used in this study to check the specificity of the loop-mediated isothermal amplification (LAMP) assay (Tp, time to positive) and the TaqMan^®^ qPCR assay (Ct). The PCR results reported the presence or absence of a 308 bp amplicon. Aflatoxin production is reported in ppm. Standard deviation was included for each Tp, Ct, melting temperature and for the four aflatoxins. ±: PCR result was not clear. Neg = negative, Pos = positive; n.d.: not detected; Melting = melting temperature.

Name	Species	PCR	LAMP Assay		qPCR TaqMan^®^ Probe (Ct)	qPCR with SYBR Green		Aflatoxin Production (ppm)			
			Tp (min:s)	Melting (°C)		Ct	Melting (°C)	AFB1	AFB2	AFG1	AFG2
Strains from Leaves											
FS2	*A. flavus*	Pos	23:21 ± 03:10	89.56 ± 0.09	25.68 ± 0.12	12.98 ± 0.0	79.79 ± 0.00	38.8 ± 0.7	2.3 ± 0.2	n.d.	n.d.
FS3	*A. flavus*	Neg	Neg	84.13 ± 0.265	Neg	15.66 ± 0.0	79.94 ± 0.21	n.d.	n.d.	n.d.	n.d.
FS4	*A. flavus*	Neg	Neg	84.03 ± 0.093	Neg	34.04 ± 5.1	64.74 ± 10.53	n.d.	n.d.	n.d.	n.d.
FS5	*A. flavus*	Neg	Neg	84.18 ± 0.086	Neg	22.60 ± 1.0	79.64 ± 0.00	n.d.	n.d.	n.d.	n.d.
FS6	*A. flavus*	Neg	Neg	84.23 ± 0.088	Neg	23.93 ± 0.6	79.34 ± 0.11	n.d.	n.d.	n.d.	n.d.
FS7	*A. flavus*	Pos	21:33 ± 01:52	89.61 ± 0.17	20.38 ± 0.46	23.10 ± 0.8	79.19 ± 0.21	8.5 ± 1.5	1.6 ± 0.5	n.d.	n.d.
FS8	*A. parasiticus*	Pos	20:47 ± 02:07	89.51 ± 0.01	20.11 ± 0.36	Neg	65.78 ± 0.42	47,491 ± 507	17,262 ± 113	75,443 ± 1698	28,789 ± 669
FS9	*A. flavus*	Pos	19:19 ± 01:10	89.21 ± 0.15	23.49 ± 0.93	37.71 ± 0.9	65.03 ± 0.74	43.2 ± 8.4	2.2 ± 0.6	n.d.	n.d.
FS10	*Aspergillus* sp.	Neg	Neg	84.13 ± 0.301	Neg	18.18 ± 0.7	79.79 ± 0.11	n.d.	n.d.	n.d.	n.d.
FV1	*A. flavus*	Pos	22:37 ± 03:27	89.51 ± 0.15	26.57 ± 0.25	Neg	Neg	238 ± 89.4	n.d.	n.d.	n.d.
FV2	*A. flavus*	Pos	20:14 ± 02:13	89.60 ± 0.08	26.79 ± 0.40	37.10 ± 0.1	65.19 ± 0.21	27.6 ± 3.4	n.d.	n.d.	n.d.
FV3	*A. flavus.*	Pos	19:43 ± 01:20	89.61 ± 0.09	25.13 ± 0.73	29.30 ± 0.2	79.19 ± 0.00	314 ± 31.3	1.8	n.d.	n.d.
FV4	*A. flavus*	Neg	Neg	84.33 ± 0.090	Neg	34.44 ± 6.7	66.82 ± 8.53	n.d.	n.d.	n.d.	n.d.
FV5	*A. flavus*	Pos	22:57 ± 02:21	89.50 ± 0.01	24.09 ± 0.40	17.20 ± 0.1	79.78 ± 0.11	46,996 ± 4177	9871 ± 288	n.d.	n.d.
FV7	*A. flavus*	Neg	Neg	84.22 ± 0.092	Neg	33.54 ± 3.4	79.34 ± 0.63	n.d.	n.d.	n.d.	n.d.
FV8	*A. flavus*	Pos	20:11 ± 04:41	89.66 ± 0.01	24.40 ± 0.27	30.18 ± 1.7	79.49 ± 0.00	133 ± 8.8	n.d.	n.d.	n.d.
FV9	*A. flavus*	Neg	Neg	84.29 ± 0.009	Neg	29.58 ± 0.4	79.34 ± 0.11	n.d.	n.d.	n.d.	n.d.
FV10	*A. flavus*	Neg	Neg	84.29 ± 0.010	Neg	12.68 ± 0.4	79.78 ± 0.00	n.d.	n.d.	n.d.	n.d.
FP1	*A. flavus*	Neg	Neg	84.08 ± 0.081	Neg	37.52 ± 0.8	66.66 ± 9.70	n.d.	n.d.	n.d.	n.d.
FP2	*A. flavus*	Pos	21:40 ± 02:28	89.61 ± 0.09	22.21 ± 0.31	30.56 ± 0.2	79.48 ± 0.11	106 ± 7.3	13.7 ± 1.5	n.d.	n.d.
FP3	*A. flavus*	Pos	21:31 ± 01:22	89.55 ± 0.09	22.40 ± 0.28	13.70 ± 0.0	79.77 ± 0.11	495 ± 101	12.9 ± 4.4	n.d.	n.d.
FP4	*Aspergillus* sp.	Pos	22:35 ± 02:25	89.66 ± 0.01	21.13 ± 0.91	14.08 ± 0.2	79.77 ± 0.11	369 ± 37.1	11.8 ± 0.6	n.d.	n.d.
FP5	*Aspergillus* sp.	Neg	Neg	84.18 ± 0.176	Neg	37.96 ± 1.7	66.34 ± 9.60	n.d.	n.d.	n.d.	n.d.
FP6	*Aspergillus* sp.	Neg	Neg	84.33 ± 0.085	Neg	15.75 ± 0.0	79.47 ± 0.00	n.d.	n.d.	n.d.	n.d.
FP8	*A. flavus*	Pos	Neg	84.24 ± 0.098	36.79 ± 0.40	34.3 ± 1.1	79.62 ± 0.00	n.d.	n.d.	n.d.	n.d.
FP9	*A. flavus*	Pos	20:52 ± 01:32	89.51 ± 0.16	22.39 ± 0.14	13.59 ± 0.0	79.93 ± 0.00	12.9 ± 1.1	2.9 ± 1	n.d.	n.d.
Strains from Bracts											
BV2	*Aspergillus* sp.	Pos	21:38 ± 01:49	89.43 ± 0.11	24.21 ± 0.69	26.00 ± 0.2	79.33 ± 0.32	493 ± 108	12.5 ± 4.1	n.d.	n.d.
BV4	*A. flavus*	Pos	22:10 ± 02:04	89.43 ± 0.31	25.47 ± 0.58	13.85 ± 0.4	79.62 ± 0.11	255 ± 51.5	2.5 ± 1.2	n.d.	n.d.
BV5	*A. flavus*	Pos	22:58 ± 02:34	89.66 ± 0.16	22.12 ± 0.51	Neg	66.36 ± 0.00	47.0 ± 7.2	1.2 ± 0.2	n.d.	n.d.
BV6	*Aspergillus* sp.	Pos	21:39 ± 02:22	89.43 ± 0.10	21.25 ± 0.38	21.91 ± 0.0	79.62 ± 0.00	34.1 ± 0.8	4.0 ± 0.8	n.d.	n.d.
BV8	*A. flavus*	Pos	21:28 ± 03:43	89.36 ± 0.25	22.84 ± 0.28	33.73 ± 4.9	66.51 ± 9.17	28.3 ± 4.8	4.5 ± 0.5	n.d.	n.d.
BV9	*A. flavus*	Pos	22:59 ± 02:46	89.41 ± 0.29	21.97 ± 0.50	24.52 ± 0.3	79.63 ± 0.11	386 ± 61.2	0.8 ± 0.3	n.d.	n.d.
BV10	*A. flavus*	Pos	Neg	84.13 ± 0.016	32.45 ± 0.63	30.12 ± 0.6	79.63 ± 0.21	n.d.	n.d.	n.d.	n.d.
BS1	*A. parasiticus*	±	26:52 ± 04:52	89.43 ± 0.11	23.68 ± 0.12	38.00 ± 0.0	65.76 ± 0.21	10,051 ± 784	15,086 ± 3774	92,163 ± 4662	38,362 ± 1360
BS3	*A. flavus*	Pos	Neg	84.13 ± 0.001	40.04 ± 0.33	12.72 ± 0.2	80.08 ± 0.00	9.5	n.d.	n.d.	n.d.
BS4	*A. flavus*	Pos	37:13 ± 00:00	88.99	30.78 ± 0.16	36.58 ± 0.3	65.76 ± 0.11	25.9 ± 2	2.8 ± 0.9	n.d.	n.d.
BS5	*A. flavus*	Pos	Neg	84.29 ± 0.014	31.72 ± 0.27	29.46 ± 0.35	80.63 ± 0.08	n.d.	n.d.	n.d.	n.d.
BS6	*A. flavus*	Neg	19:35 ± 02:14	89.51 ± 0.21	22.01 ± 0.45	Neg	64.72 ± 0.53	10,011 ± 322	2264 ± 173	n.d.	n.d.
BS7	*Aspergillus* sp.	Neg	Neg	84.20 ± 0.11	Neg	Neg	64.70 ± 0.42	n.d.	n.d.	n.d.	n.d.
BS8	*A. sojae*	Neg	Neg	84.13 ± 0.22	Neg	34.15 ± 0.3	79.62 ± 0.11	n.d.	n.d.	n.d.	n.d.
BS9	*A. flavus*	Neg	22:30 ± 02:54	89.41 ± 0.21	20.92 ± 0.25	Neg	65.30 ± 0.74	23.5 ± 4.7	1.9 ± 0.4	n.d.	n.d.
BS10	*A. flavus*	Neg	Neg	84.06 ± 0.09	Neg	Neg	65.31 ± 0.74	n.d.	n.d.	n.d.	n.d.
Strains from Kernel											
NP1	*A. flavus*	Pos	Neg	Neg	27.80 ± 11.94	33.15 ± 0.40	78.28 ± 0.00	n.d.	n.d.	n.d.	n.d.
NP2	*Aspergillus* sp.	Neg	Neg	Neg	26.08 ± 0.94	22.41 ± 0.15	78.51 ± 0.11	n.d.	n.d.	n.d.	n.d.
NP3	*A. flavus*	Neg	Neg	Neg	Neg	Neg	Neg	n.d.	n.d.	n.d.	n.d.
NP4	*A. flavus*	Pos	29:17 ± 00:22	87.34 ± 0.09	28.55 ± 0.46	23.58 ± 0.14	80.74 ± 3.26	7.3 ± 1.3	2.5 ± 1.2	n.d.	n.d.
NP5	*A. flavus*	Pos	22:53 ± 02:08	88.97 ± 0.00	21.07 ± 1.85	16.65 ± 0.13	78.66 ± 0.11	137 ± 1.6	n.d.	n.d.	n.d.
NP6	*A. flavus*	Pos	Neg	Neg	34.03 ± 0.17	29.22 ± 0.47	78.58 ± 0.21	n.d.	n.d.	n.d.	n.d.
NP7	*A. flavus*	Neg	Neg	Neg	Neg	Neg	Neg	n.d.	n.d.	n.d.	n.d.
NP8	*A. flavus*	Pos	22:40 ± 02:43	89.22 ± 0.15	22.79 ± 0.95	17.11 ± 0.13	78.80 ± 0.11	46.9 ± 4.6	45.7 ± 0.1	n.d.	n.d.

**Table 2:** Strains from chestnuts used in this study to check the specificity of the LAMP assay (Tp, time to positive) and the TaqMan^®^ qPCR assay (Ct). The PCR results reported the presence or absence of a 308 bp amplicon. Aflatoxin production is reported in ppm. Standard deviation was included for each Tp, Ct, melting temperature and for the four aflatoxins. Neg = negative, Pos = positive; n.d.: not detected; Melting = melting temperature.

Strain	Species	PCR Result	LAMP Assay		qPCR	qPCR with SYBR Green		Aflatoxin Production (ppm)			
			Tp (min:s)	Melting (°C)	TaqMan^®^	Ct	Melting (°C)	AFB1	AFB2	AFG1	AFG2
					Probe (Ct)						
XN	*A. flavus*	Neg	Neg	Neg	Neg	Neg	Neg	n.d.	n.d.	n.d.	n.d.
AF2	*A. flavus*	Neg	Neg	Neg	Neg	Neg	66.68 ± 1.26	n.d.	n.d.	n.d.	n.d.
AF3	*A. flavus*	Neg	Neg	84.14 ± 0.150	Neg	30.20 ± 0.0	79.34	n.d.	n.d.	n.d.	n.d.
AF4	*A. flavus*	Neg	Neg	84.24 ± 0.089	Neg	Neg	67.57 ± 1.05	n.d.	n.d.	n.d.	n.d.
AF11	*A. flavus*	Neg	Neg	Neg	Neg	Neg	65.93 ± 2.21	n.d.	n.d.	n.d.	n.d.
AF12	*A. flavus*	Neg	Neg	84.29 ± 0.012	Neg	Neg	67.12 ± 0.42	n.d.	n.d.	n.d.	n.d.
AF17	*A. flavus*	Neg	Neg	84.29 ± 0.006	Neg	30.58 ± 1.2	79.04	n.d.	n.d.	n.d.	n.d.
AF18	*A. flavus*	Neg	Neg	84.30 ± 0.000	Neg	37	65.79 ± 10.22	n.d.	n.d.	n.d.	n.d.
AF20	*A. flavus*	Neg	Neg	84.20 ± 0.174	Neg	Neg	65.49	n.d.	n.d.	n.d.	n.d.
AF24	*A. flavus*	Neg	Neg	Neg	Neg	27.10 ± 0.5	79.34	n.d.	n.d.	n.d.	n.d.
AFCAL2	*A. flavus*	Pos	23:12 ± 00:30	89.66 ± 0.01	22.92 ± 1.55	15.33 ± 0.6	80.08 ± 0.00	152 ± 26	4.3 ± 0.3	n.d.	n.d.
AFCAL3	*A. flavus*	Pos	25:09 ± 04:12	89.67 ± 0.01	26.34 ± 0.52	18.95 ± 0.8	80.23 ± 0.21	14.2 ± 2.3	n.d.	n.d.	n.d.
AFCAL4	*A. flavus*	Pos	22:16 ± 03:12	89.82 ± 0.26	20.44 ± 0.98	13.58 ± 0.3	80.08 ± 0.11	16.1 ± 1.4	n.d.	n.d.	n.d.
AFCAL6	*A. flavus*	Pos	21:03 ± 03:13	89.51 ± 0.01	18.28 ± 0.67	11.88 ± 0.3	80.23 ± 0.11	2.5 ± 0.2	n.d.	n.d.	n.d.
AFCAL7	*A. flavus*	Pos	22:30 ± 00:33	89.67 ± 0.01	26.00 ± 1.67	18.35 ± 0.0	80.08 ± 0.00	n.d.	n.d.	n.d.	n.d.
AFCAL9	*A. flavus*	Neg	Neg	89.36 ± 2.93	Neg	32.08 ± 3.4	79.78 ± 0.00	n.d.	n.d.	n.d.	n.d.
AFLX1	*A. flavus*	Neg	Neg	84.15 ± 0.151	Neg	30.60 ± 0.1	79.78	150 ± 25.4	6.0 ± 0.1	n.d.	n.d.
AFLX2	*A. flavus*	Neg	Neg	84.15 ± 0.150	Neg	Neg	65.33	43.1 ± 12.9	4.4 ± 0.9	n.d.	n.d.
AFLX5	*A. flavus*	Neg	Neg	84.30 ± 0.006	Neg	Neg	64.43 ± 0.32	n.d.	n.d.	n.d.	n.d.
AFLX7	*A. flavus*	Pos	22:14 ± 03:20	89.56 ± 0.09	21.89 ± 0.74	Neg	Neg	n.d.	n.d.	n.d.	n.d.
AFLX8	*A. flavus*	Pos	21:14 ± 04:38	89.61 ± 0.09	33.22 ± 0.83	31.03 ± 0.3	79.63 ± 0.00	817 ± 128	201 ± 39.8	n.d.	n.d.
AFLX9	*A. flavus*	Neg	Neg	84.14 ± 0.158	Neg	32.61 ± 8.0	66.07	n.d.	n.d.	n.d.	n.d.
AFLX10	*A. flavus*	Pos	Neg	84.12 ± 0.10	Neg	26.92	61.31 ± 3.89	n.d.	n.d.	n.d.	n.d.
AFSP1	*A. flavus*	Neg	Neg	84.29 ± 0.006	Neg	32.88 ± 2.9	79.34	n.d.	n.d.	n.d.	n.d.
AFSP2	*A. flavus*	Neg	Neg	84.35 ± 0.377	Neg	38.72 ± 1.5	66.97 ± 0.95	66.97 ± 0.95	5.6 ± 0.7	n.d.	n.d.
AFSP3	*A. flavus*	Neg	Neg	84.20 ± 0.174	39.56 ± 0.65	28.96 ± 0.0	79.34	5.9 ± 0.1	n.d.	n.d.	n.d.
AFSP4	*A. flavus*	Pos	21:38 ± 02:40	89.61 ± 0.08	25.59 ± 0.42	16.34 ± 0.3	79.79 ± 0.32	177.5 ± 8.9	4.1 ± 1.1	n.d.	n.d.
AFSP5	*A. flavus*	Neg	Neg	84.29 ± 0.142	Neg	Neg	63.09 ± 0.84	n.d.	n.d.	n.d.	n.d.
AFSP6	*A. flavus*	Pos	20:58 ± 03:19	89.62 ± 0.08	23.08 ± 0.40	13.25 ± 1.0	79.19 ± 0.21	54.7 ± 4.2	1.9 ± 0.8	n.d.	n.d.
AFSP7	*A. flavus*	Pos	23:13 ± 01:36	89.72 ± 0.22	29.83 ± 0.76	22.47 ± 2.7	79.64 ± 0.11	11.2 ± 0.5	n.d.	n.d.	n.d.
AFCAL1	*A. oryzae* var *effusus*	Neg	Neg	84.29 ± 0.01	Neg	37.9	80.97	n.d.	n.d.	n.d.	n.d.
AFCAL5	*A. oryzae* var *effusus*	Neg	Neg	84.24 ± 0.08	Neg	Neg	65.47 ± 0.21	n.d.	n.d.	n.d.	n.d.
AFCAL10	*A. oryzae* var *effusus*	Neg	Neg	84.29 ± 0.006	Neg	Neg	66.07 ± 0.74	n.d.	n.d.	n.d.	n.d.
AFLX3	*A. oryzae* var *effusus*	Neg	Neg	84.29 ± 0.006	Neg	31.87	79.64	n.d.	n.d.	n.d.	n.d.
AFLX4	*A. oryzae* var *effusus*	Neg	Neg	84.24 ± 0.080	Neg	Neg	64.88 ± 0.21	n.d.	n.d.	n.d.	n.d.
MCAL1	*A. tamarii*	Neg	Neg	84.14 ± 0.006	Neg	28.67 ± 0.3	79.63	n.d.	n.d.	n.d.	n.d.
MCAL2	*A.tamarii*	Neg	Neg	84.29 ± 0.004	Neg	34.99 ± 4.5	79.48 ± 9.38	n.d.	n.d.	n.d.	n.d.
AFCAL8	*A. parasiticus*	Pos	24:10 ± 04:20	89.67 ± 0.15	21.72 ± 1.33	16.52 ± 0.3	79.93 ± 0.21	352 ± 59.4	68.2 ± 27.5	944 ± 169	128 ± 25.9
AFLX6	*A. parasiticus*	Pos	20:58 ± 03:31	89.57 ± 0.08	22.66 ± 0.87	18.58 ± 0.3	79.44 ± 0.28	387 ± 36.7	42.1 ± 8.8	662 ± 8.8	108 ± 10.1
AFSP8	*A. toxicarius*	Pos	27:49 ± 00:37	89.59 ± 0.11	28.56 ± 0.41	19.24 ± 0.1	79.64 ± 0.21	910 ± 78.2	235 ± 24.2	1142 ± 78.0	392 ± 59.2

**Table 3:** PCR, LAMP and qPCR primers designed and used in this study. Hyphens denote the division between F1 and F2 and B1 and B2 of the FIP and BIP (forward and backward inner) primers.

Name	Sequence	Technique
FIP	5′-CGAGAATGCTAAGACAGCTGCAAATTAG-CTGGTGGCATATAATGCCGC-3′	LAMP
BIP	5′-CGCCGTCGGGATCGAATGGAG-CAAAGTTAGCCGGGTACTATCG-3′	
loopF	5′-GCCACCTGAAAGACGTCCAGCA-3′	
loopB	5′-AAGCGTTAAGCAGAACAGGTAACG-3′	
F3	5′-CGGAGTACATGGACCAGGTG-3′	
B3	5′-AGAAGACAGAAGAAAAGGTAAGA-3′	
COX-FIP	5′-ATGGATTTGRCCTAAAGTTTCAGGGCAGGATTTCACTATTGGGT-3′	LAMP [52]
COX-BIP	5′-TGCATTTCTTAGGGCTTTCGGATCCRGCGTAAGCATCTG-3′	
COX-loopF	5′-ATGTCCGACCAAAGATTTTACC-3′	
COX-loopB	5′-GTATGCCACGTCGCATTCC-3′	
COX-F3	5′-TATGGGAGCCGTTTTTGC-3′	
COX-B3	5′-AACTGCTAAGRGCATTCC-3′	
AflF	5′-CTGGACGTCTTTCAGGT G-3′	qPCR
AflR	5′-CTTCTCCATTCGATCCCG-3′	
Afl-probe	5′6-Fam-AM- CCTA ATT TGC AGC TGT CTT AGC ATT CTC- ZNA-4-Eclipse-3′	
AfF	5′- CCAGGTCTCGATCCAGAGGC -3′	PCR
AfR	5′- CCGGAACGTGAACTTATGTGCAC -3′	

**Table 4:** Sensitivity testing of the LAMP assay using the DNA of two strains (*A. flavus* strain FS7 and *A. parasiticus* strain AFLX6) at different concentrations. Standard deviation was included for each Tp and melting temperature. Tp = time to positive; Melting = melting temperature; n°P = number of positive technical replicates out of 3.

	*A. flavus* FS7			*A. parasiticus* AFLX6		
DNA Concentration	Tp (min:s)	Melting (°C)	n°P	Tp (min:s)	Melting (°C)	n°P
10–50 ng/µL	22:49 ± 00:52	89.51 ± 0.00	3	20:57 ± 03:31	89.57 ± 0.08	3
1–10 ng/µL	25:04 ± 00:21	89.61 ± 0.09	3	22:50 ± 02:07	89.67 ± 0.15	3
100–999 pg/µL	31:37 ± 07:39	89.52 ± 0.26	3	28:22 ± 03:08	89.61 ± 0.08	3
10–99 pg/µL	Negative	Negative	0	Negative	Negative	0

**Table 5:** Results of the LAMP assay using crude DNA extracts and DNA extracted with a commercial kit of artificially inoculated hazelnuts at different concentrations with the conidia of three *Aspergillus* strains (AFSP1, non-aflatoxigenic *A. flavus*, AFSP4, aflatoxigenic *A. flavus* and AFLX6, aflatoxigenic *A. parasiticus*). The crude DNA extraction reliability was evaluated using the COX LAMP assay as internal control. The TaqMan^®^ qPCR was used to calculate the number of cells present in each hazelnut. For each conidial concentration, 3 hazelnuts were inoculated. Tp: LAMP time to positive; Melting: LAMP melting temperature; Neg: negative. n°P = number of positive technical replicates out of 3.

Conidial Concentration	Crude DNA Extraction				COX Assay		Commercial Kit DNA Extraction					
	Tp (min:s)	Melting (°C)	n°P	Cells/Hazelnut (qPCR)	Tp (min:s)	Melting (°C)	Tp (min:s)	Melting (°C)	n°P	TaqMan (Ct)	n°P	Cells/µL (qPCR) (Range of Cells/uL)
**AFSP1 (non-aflatoxigenic *A. flavus*)**												
3.09 × 10^5^	Neg	Neg	0	Neg	05:43 ± 00:01	85.21 ± 0.00	Neg	Neg	0	Neg	0	Neg
3.09 × 10^6^	Neg	Neg	0	Neg	05:45 ± 00:49	85.22 ± 0.00	Neg	Neg	0	Neg	0	Neg
3.09 × 10^7^	Neg	Neg	0	Neg	05:09 ± 00:42	85.36 ± 0.00	Neg	Neg	0	Neg	0	Neg
AFSP4 (aflatoxigenic *A. flavus*)												
3.03 × 10^5^	45:07 ± 12:58	88.91 ± 0.15	3	1.7 × 10^5^	09:16 ± 05:33	85.27 ± 0.09	38:34 ± 06:39	88.51 ± 0.28	9	30.3 ± 3.2	9	5.8 × 10^3^ (2.6 × 10^4^–53)
3.03 × 10^6^	39:31 ± 00:00	89.21 ± 0.00	1	1.1 × 10^4^	05:39 ± 00:44	85.21 ± 0.00	42:53 ± 04:47	88.65 ± 0.09	9	31.6 ± 1.7	9	355 (640–16)
3.03 × 10^7^	43:38 ± 05:25	89.01 ± 0.18	3	6.3 × 10^5^	08:18 ± 05:36	85.27 ± 0.09	32:59 ± 02:26	88.51 ± 0.30	9	26.7 ± 1.7	9	2.1 × 10^4^ (6.2 × 10^4^–2.8 × 10^3^)
AFLX6 (aflatoxigenic *A. parasiticus*)												
1.79 × 10^5^	44:35 ± 00:00	88.77 ± 0.00	1	5.6 × 10^5^	05:08 ± 00:26	85.14 ± 0.10	34:36 ± 04:07	88.46 ± 0.12	6	32.3 ± 5.0	9	1.9 × 10^4^ (7.9 × 10^4^–8)
1.79 × 10^6^	45:26 ± 07:20	88.70 ± 0.75	2	8.4 × 10^4^	06:37 ± 00:47	85.12 ± 0.17	39:56 ± 03:31	88.46 ± 0.26	8	33.2 ± 3.2	9	2.8 × 10^3^ (9.5 × 10^3^–60)
1.79 × 10^7^	Neg	Neg	0	2.4 × 10^6^	05:48 ± 00:39	85.22 ± 0.15	33:36 ± 02:18	88.48 ± 0.18	9	27.6 ± 2.0	9	8.2 × 10^4^ (1.9 × 10–4.0 × 10^3^)

**Table 6:** Naturally contaminated hazelnuts used to evaluate the reliability of the TaqMan^®^ qPCR assay. The results listed the Ct, the number of cells in each hazelnut calculated with the TaqMan^®^ qPCR and the number of positive replicates out of three. Standard deviation was included for each Ct and for the approximate number of cells. n°P = number of positive technical replicates out of 3 with the qPCR.

Hazelnut Number	Ct	Number of Cells	n°P
1	38.83 ± 0.83	20 ± 11	3
7	38.39 ± 0.65	25 ± 11	2
8	39.35 ± 0.27	12 ± 2	2
10	37.05 ± 0.20	62 ± 8	3
22	39.15 ± 0.19	14 ± 2	2
23	39.74	8	1
32	37.35 ± 1.55	66 ± 60	2
33	37.10 ± 0.23	60 ± 10	3
34	34.35 ± 0.61	439 ± 175	3
35	34.40 ± 0.48	204 ± 68	3
36	35.44 ± 0.97	227 ± 169	3
38	35.52 ± 1.17	219 ± 139	3
39	36.25 ± 2.09	182 ± 163	3
40	36.61 ± 0.66	90 ± 38	3
41	35.52 ± 0.28	183 ± 36	3
42	36.04 ± 1.24	165 ± 153	3
43	35.91 ± 0.42	142 ± 40	3
45	39.14 ± 0.79	16 ± 8	3
47	38.71	19	1

**Table 7:** Summary of the comparison of the diagnostic sensitivity and specificity of the end-point PCR, LAMP and qPCR with SYBR Green and TaqMan ^®^ probe using DNA of 97 *Aspergillus* spp.

	PCR	LAMP	qPCR with SYBR Green	qPCR with TaqMan^®^ Probe
+LR	4.85	22	1.36	5.59
	Approximate 1 in 1.2 positive results are aflatoxigenic strains	Approximate 1 in 1.0 positive results are aflatoxigenic strains	Approximate 1 in 1.8 positive results are aflatoxigenic strains	Approximate 1 in 1.2 positive results are aflatoxigenic strains
−LR	0.16	0.11	0.6	0.13
	Approximate 1 in 1.2 negative results are non-aflatoxigenic strains	Approximate 1 in 1.1 negative results are non-aflatoxigenic strains	Approximate 1 in 1.5 negative results are non-aflatoxigenic strains	Approximate 1 in 1.1 negative results are non-aflatoxigenic strains
Diagnostic sensitivity	87.2%	89.4%	71.7%	89.4%
Diagnostic specificity	82.0%	96%	47.1%	84%

## Supplementary Materials

The following are available online at https://www.mdpi.com/2072-6651/12/12/757/s1, Figure S1: Phylogenetic tree based on the calmodulin gene sequences constructed with Maximum Likelihood method using K80 model plus gamma distribution model. Sequences from different species belonging to the *Aspergillus* section *Flavi,* identified by Frisvad et al. [4] were included as references, Figure S2: LAMP, qPCR and end-point PCR primers alignment with the *aflT* gene within the aflatoxin biosynthetic cluster, Figure S3: LAMP, qPCR and end-point PCR primers alignment with the *aflT* gene of *A. flavus* (GenBank AF515601.1) showing the exons and introns; Figure S4: Agarose gel of the 308 bp amplicon obtained with primers AfF and AfR of the 97 *Aspergillus* spp. used in this study, Figure S5: Agarose gel showing the limit of detection of the 308 bp PCR with the primers AfF and AfR. 1: GelPilot Wide Range Ladder (QIAGEN); 2: GelPilot 100 bp Ladder (QIAGEN), 3: *A. flavus* FV3 (110 ng/µL); 4: *A. flavus* FV3 (11 ng/µL); 5: *A. flavus* FV3 (1.1 ng/µL); 6: *A. flavus* FV3 (110 pg/µL); 7: *A. flavus* FV3 (11 pg/µL); 8: *A. flavus* FV3 (1.1 pg/µL); 9: *A. flavus* FV3 (110 fg/µL); 10: *A. flavus* FV3 (11 fg/µL); 11: Negative Control with water; 12: GelPilot 100 bp Ladder (QIAGEN); 13: GelPilot Wide Range Ladder (QIAGEN), Figure S6: LAMP amplifications exported from the Genie II® instrument (A), and melting peaks (B) of 10-fold dilutions of the DNA of *A. flavus* FS7 strain. The amplification was confirmed by running an agarose gel of one of the three replicates of each dilution (C), Figure S7: qPCR standard curves with SYBR Green (A), and TaqMan ® probe standard curves using the DNA of *A. flavus* (FS7) (B) and *A. parasiticus* (AFLX6) (C), Table S1: Reference strains used in this study to check the specificity of the LAMP assay (Tp, time to positive) and the qPCR assays (Ct). The PCR results reported the presence or absence of a 308 bp-amplicon. Aflatoxin production is reported in ppm. Neg = negative, Pos = positive; n.d.: not detected; Melting: melting temperature, Table S2: Other species used in the specificity panel of the LAMP assay (Tp, time to positive) and the qPCR assay (Ct). The PCR results reported the presence or absence of a 308 bp-amplicon. Standard deviation was included for each Tp and melting temperature. Tp: time to positive. Anneal: melting temperature, Table S3: Reference strains used for the identification of the hazelnut isolates. The strains were previously identified by Frisvad et al. [4]. GenBank accession number report the sequence for the calmodulin used to infer a phylogenetic tree that identifies the hazelnut isolates.
