# Supplementary material for: Development of PCR, LAMP and qPCR Assays for the Detection of Aflatoxigenic Strains of Aspergillus flavus and A. parasiticus in Hazelnut
Source: Toxins (Basel). 2020 Nov 30;12(12):757. doi: 10.3390/toxins12120757 (PMC7761073; doi:10.3390/toxins12120757)
Supplement: Supplementary file 1 [file toxins-12-00757-s001.pdf]

Supplementary Materials: Development of PCR, LAMP and qPCR Assays for the Detection of Aflatoxigenic Strains of *Aspergillus flavus* and *A. parasiticus* in Hazelnut

Sara Franco Ortega, Ilenia Siciliano, Simona Prencipe, Maria Lodovica Gullino and Davide Spadaro

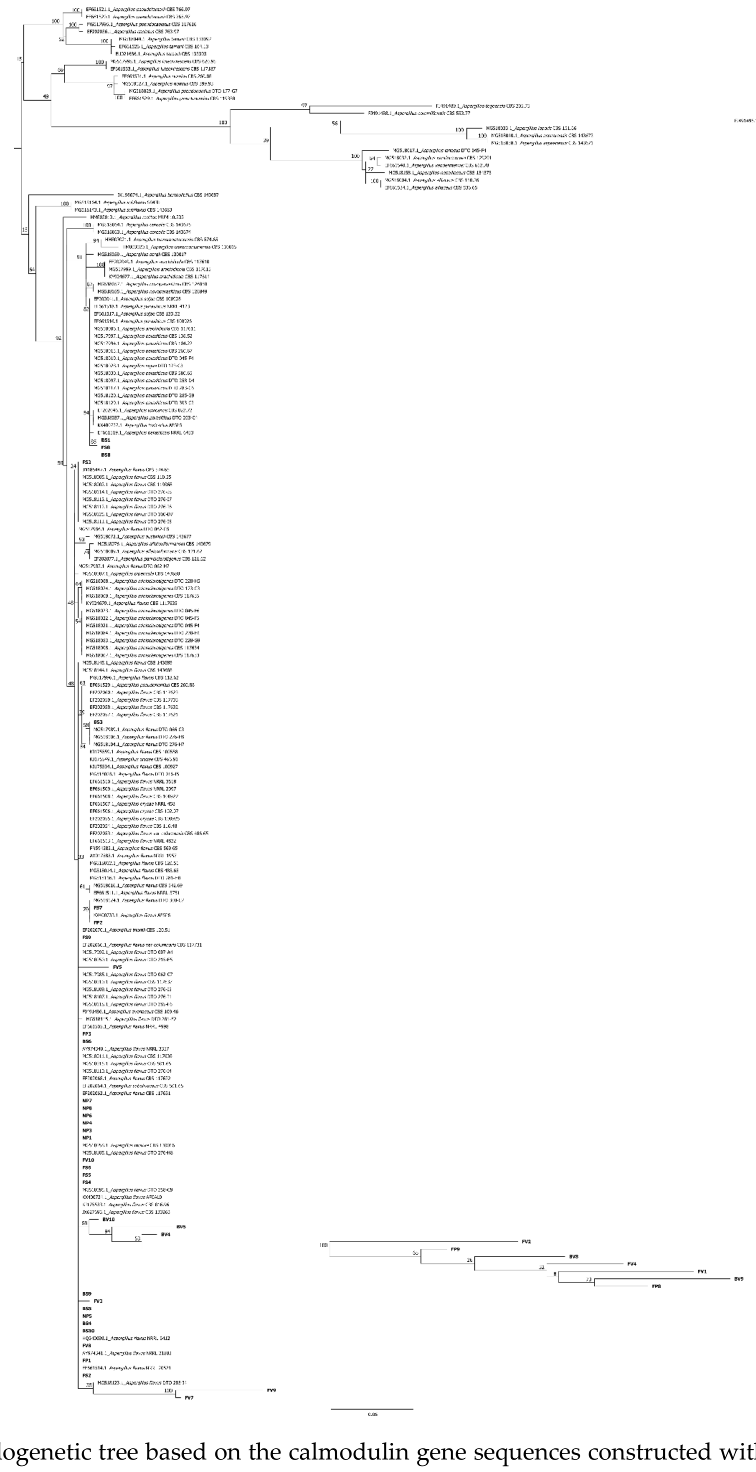

Figure S1. Phylogenetic tree based on the calmodulin gene sequences constructed with Maximum Likelihood method using K80 model plus gamma distribution model. Sequences from different species belonging to the *Aspergillus* section *Flavi*, identified by Frisvad et al. [4] were included as references.

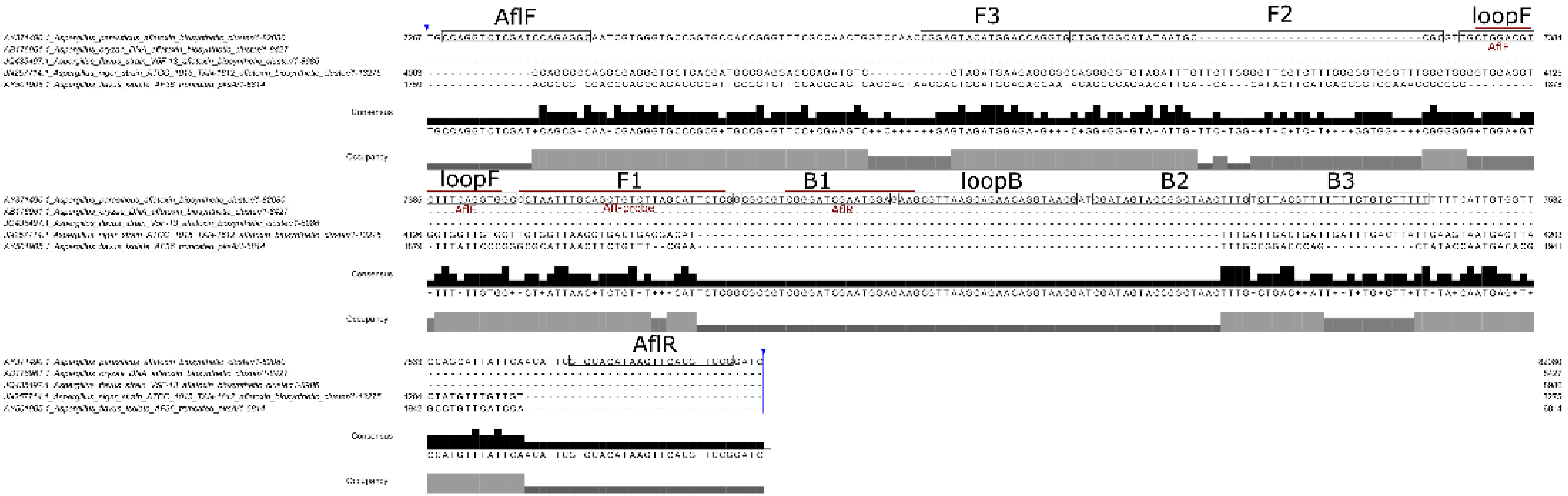

Figure S2. LAMP, qPCR and end-point PCR primers alignment with the *aflT* gene within the aflatoxin biosynthetic cluster.

> *aflT A. flavus* (GenBank: AF515601.1

tttctttctttctctctctctctcttttctttctcatccccttcttcattgttatcaagttgttaatcattcctgtacctcctttatatccttgaccccgttccccttccccctctccgc
aaaacaacaccagactcttctctccacccagcaccatcgtccagggggtccgagtatgcagagcaataaggagacggcactcggccccgagcgcaaccccac
gggcgagggggaggtgcggacatctaacgaccat gacatgctaatcgacgaggctgccgacgcgagtagtcacatctccggcatgaagttgtatctcattgt
gctaagtctattgctcgctgtgttctgcgtggctctcgataacaccgttcgttcccctggcgctgttcttcctctttcctggcatggcgggttttgaactgatgatgatt
gtggcgcagatcctgtccgttgccatccctcgcatcacggatgaattccaccggctgaacgatattggatggtatgcg tccgcctatctactgactacgtgtggta
agtccgcgcgagatacttcttatactaaacctcgaattgccattccactgacgaccggcgggggaggacag ccttccaattactatatggcaagttatatgccctc
ttcagcaccaaatgggtctttctcgtcgccctgggtattttcgaagtgggctctctgatctgcggcgtcgcaccgtcgtccgtagttctcatcgtcggccgtgccatc
gcgggagtggggagctcggggatctttacaggtgctctggtcaccatgcggcatattgtgcccttggccaagcgaccagtttacatggggctcttgggggg
atgtacgggattgcgtcagtggcaggccctctggtaagtcaccgttgatgcacccggcggctgtgacgggcaccattaaccgttccttgtgaatcgcag ttggg
gggcgccttcacgaacgaagtgacctggcgatggtgcttctacatcaatcttccggtcggaggg gtcaccgctgtggttatcctgtttcttcttcggatccccaaa
tcggcggacctgcgcacgcatggcgcctgggagatgttgaaaggcttagatccgttaggcacgattgtcttcaccccgtccatcatctgcgttctcctggcgctc
cagtggggtggggtggactacgcttggtcgaatggacgcattatcgcgctgtttgttctcttcggcgtgctcctcatcacatttatcgtcattcaggtcctcatgaa
ggataacgccaccggtatggtattcctccgttggtttccatccgttctgtaagcctcgctcaccactcccgccag taccgatcaaagtagcctctcagcgatccgta
gcatgcgcctccgtcttcgtcttcttcatcggcgcctccatgttcgtgatgatctactatgtgccgatctgg gtaacctgcctctccctagtcccctccctgtgtgatgt
gtcgcaccacgccacgatattgctaatgcatatccaatag ttccaagccattcgcaaccaatcacccgtgcaggcaggcattgattctattgccttgattttggcca
ataccgccggtgccatcatctctggcgccgtgaccaataagacagggcactacgcccactggtttattgtcagtagcgtgatcatgtcgattggcgccggctgcc
ttaccctcttcaccgtggatattgcccaatccaaatggatcggttttctcttcctctatgggatcggcgtcggcttcggctttca gcaaggcgccgtggccgttcag
gccgtgcttcccatggcacaagtccccatcggaaccgcactaatctggtttgtgcagatgttgggcggtgcgctcttcacctcggtagcgcaaaacatttcagc
acccacctagcggagaacctcgcgaacctgcagctgccaggtctcgatccagaggcaatcgtgggtgccggtgctaccgggtttcgccaactggtccaac cg
gagtacatggaccaggtgctggtggcatataatgccgcgttgctggacgtctttcaggtggccctaatttgcagctgtcttagcattctcggcgccgtcgggatc
gaatggagaagcgttaagcagaacaggtaacgaccgatagtacccggctaactttgtcttaccttttcttctgtcttcttttttcattgtggttccagcattattcaa
cattcgtgcacataagttcacgttccggatcatcggagacccgttctcatctcccttcctgcaagggagcgcgaggaccccgagcctgccatgaaaaggaacga
tgtttatttcttttaattcttgtcaaacggcttcggcgttacctcgttcaaccgcgggtcccttatttgatttccacaaatgtataatcaacatcatctatcagcgtatgt
agtactcgcgc

**FIP**	5'-CGAGAATGCTAAGACAGCTGCAAATTAG -CTGGTGGCATATAATGCCGC -3'<br>RC: GCGGCATTATATGCCACCAG -CTAATTTGCAGCTGTCTTAGCATTCTCG	
**BIP**	5'-CGCCGTCGGGATCGAATGGAG -CAAAGTTAGCCGGGTACTATCG -3'<br>RC **CGATAGTACCCGGCTAACTTTG** -CTCCATTCGATCCCGACGGCG	
**loopF**	5'-GCCACCTGAAAGACGTCCAGCA-3'<br>RC: TGCTGGACGTCTTTCAGGTGGC	**LAMP**
**loopB**	5'-AAGCGTTAAGCAGAACAGGTAACG-3'	
**F3**	5'-CGGAGTACATGGACCAGGTG -3'	
**B3**	5'-AGAAGACAGAAGAAAAGGTAAGA-3'<br>RC: **TCTTACCTTTTCTTCTGTCTTCT**	
**AflF**	5'-CTGGACGTCTTTCAGGTG-3'	
**AflR**	5'-CTTCTCCATTCGATCCCG-3'<br>RC: CGGGATCGAATGGAGAAG	**qPCR**
**Afl-probe**	5'6-Fam-AM- CCTAATTTGCAGCTGTCTTAGCATT CTC- ZNA-4-Eclipse-3'	
**AfF**	5'- CCAGGTCTCGATCCAGAGGC -3'	
**AfR**	5'- CCGGAACGTGAACTTATGTGCAC -3'<br>RC: GTGCACATAAGTTCACGTTCCGG	**PCR**

Coding regions

Figure S3. LAMP, qPCR and end-point PCR primers alignment with the aflT gene of A. flavus (GenBank AF515601.1) showing the exons and introns.

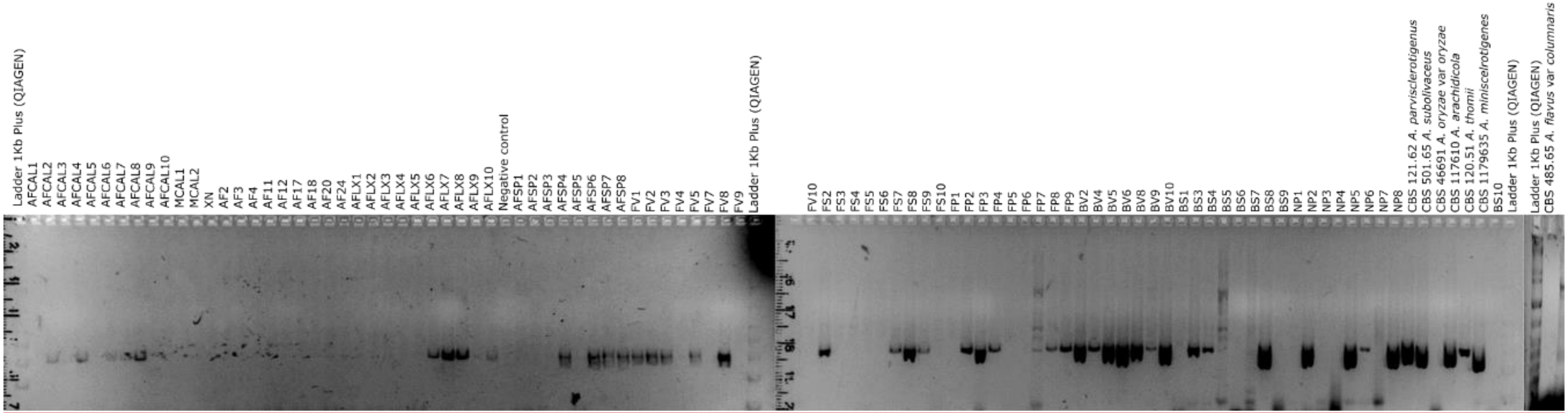

Figure S4. Agarose gel of the 308 bp amplicon obtained with primers AfF and AfR of the 97 *Aspergillus* spp. used in this study.

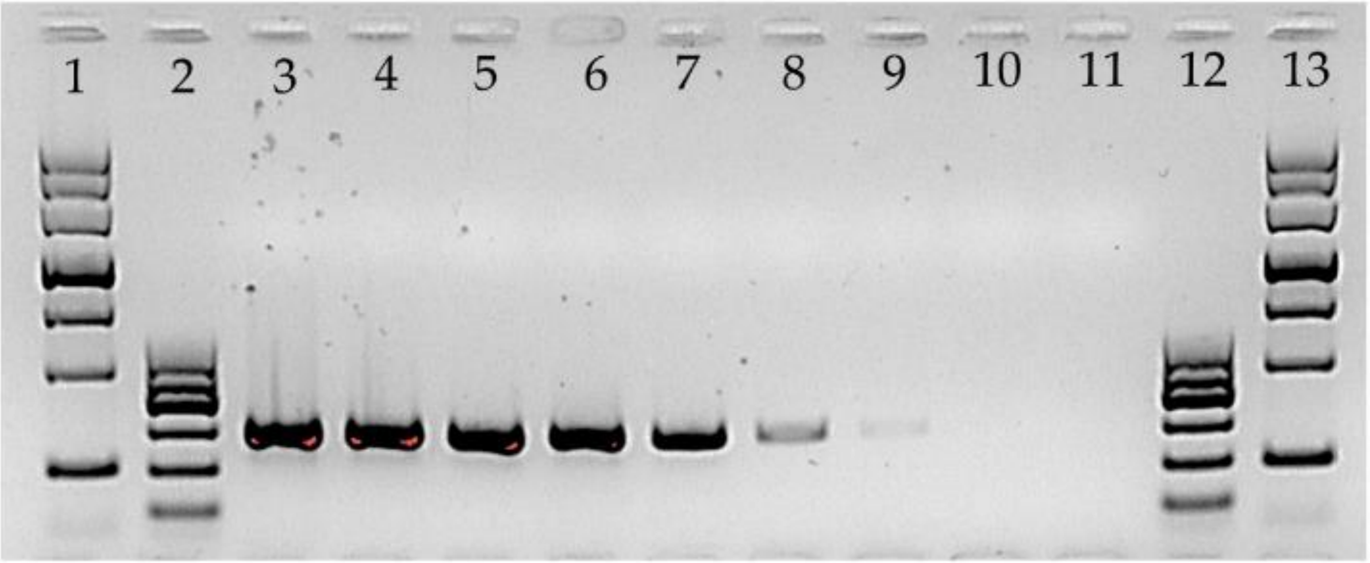

Figure S5. Agarose gel showing the limit of detection of the 308 bp PCR with the primers AfF and AfR. 1: GelPilot Wide Range Ladder (QIAGEN); 2: GelPilot 100 bp Ladder (QIAGEN), 3: *A. flavus* FV3 (110 ng/µL); 4: *A. flavus* FV3 (11 ng/µL); 5: *A. flavus* FV3 (1.1 ng/µL); 6: *A. flavus* FV3 (110 pg/µL); 7: *A. flavus* FV3 (11 pg/µL); 8: *A. flavus* FV3 (1.1 pg/µL); 9: *A. flavus* FV3 (110 fg/µL); 10: *A. flavus* FV3 (11 fg/µL); 11: Negative Control with water; 12: GelPilot 100 bp Ladder (QIAGEN); 13: GelPilot Wide Range Ladder (QIAGEN).

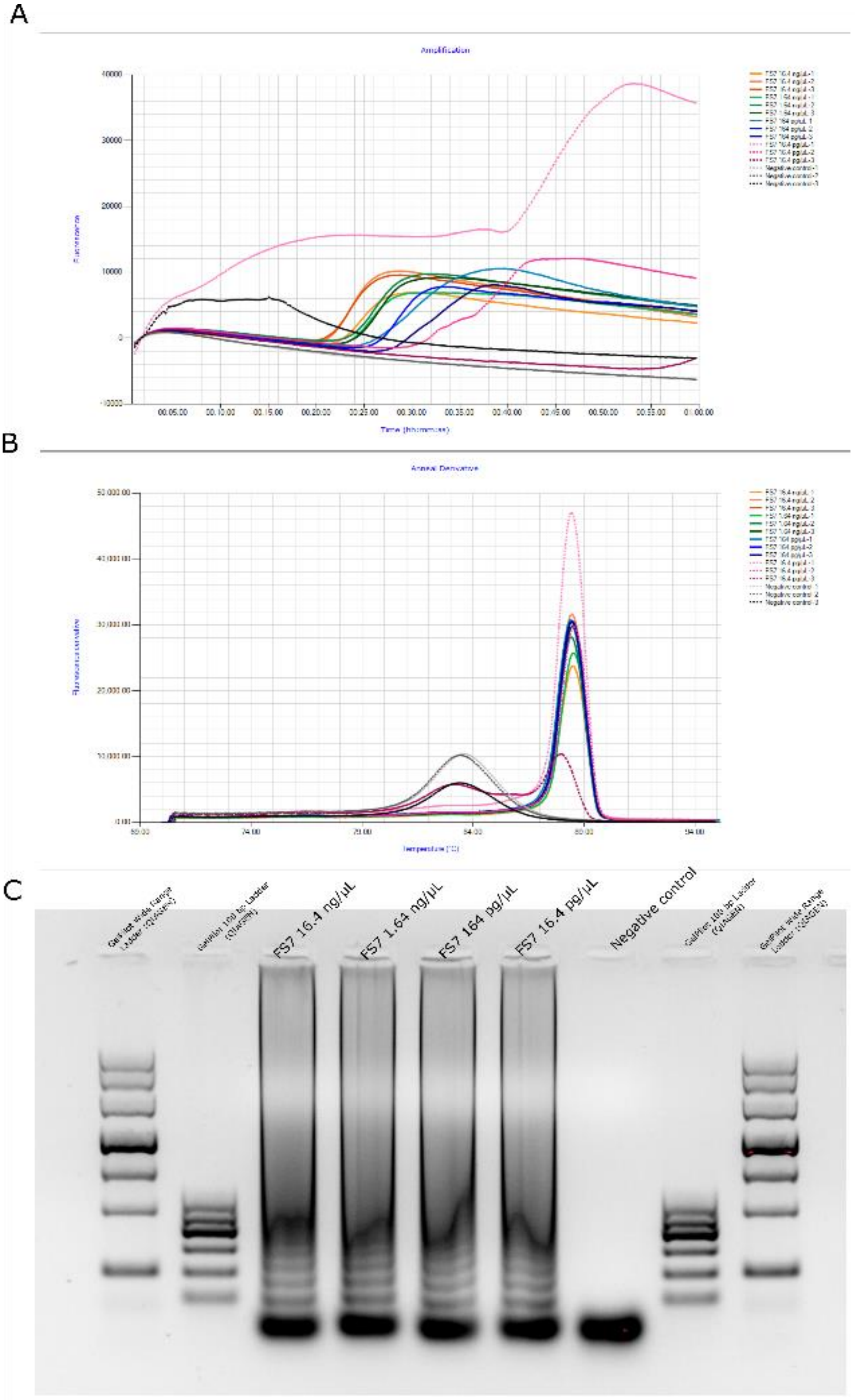

Figure S6. LAMP amplifications exported from the Genie II® instrument (**A**), and melting peaks (**B**) of 10-fold dilutions of the DNA of *A. flavus* FS7 strain. The amplification was confirmed by running an agarose gel of one of the three replicates of each dilution (**C**).

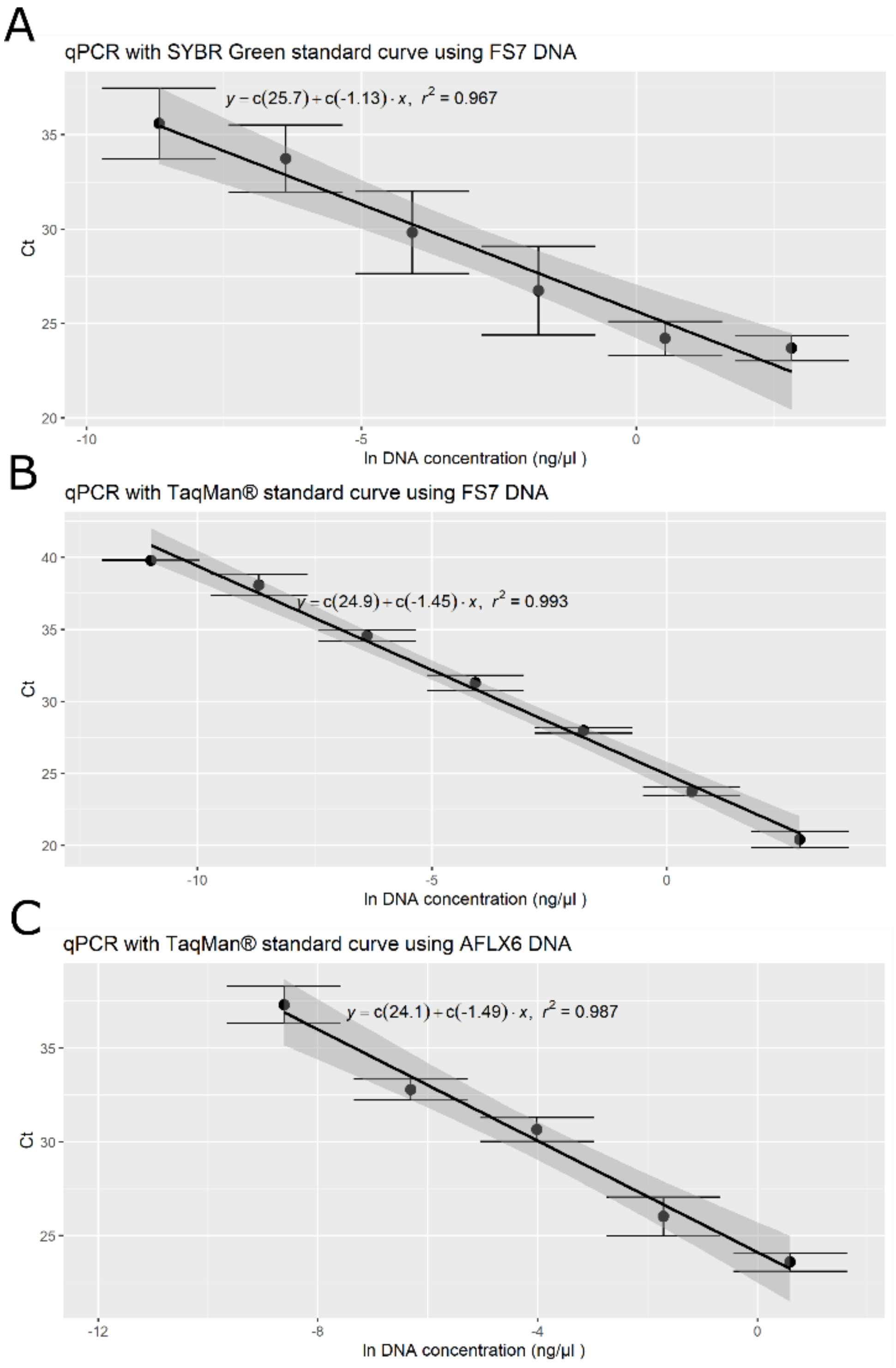

Figure S7. qPCR standard curves with SYBR Green (**A**), and TaqMan ® probe standard curves using the DNA of *A. flavus* (FS7) (**B**) and *A. parasiticus* (AFLX6) (**C**).

Table S1. Reference strains used in this study to check the specificity of the LAMP assay (Tp, time to positive) and the qPCR assays (Ct). The PCR results reported the presence or absence of a 308 bp-amplicon. Aflatoxin production is reported in ppm. Neg=negative, Pos=positive; n.d.: not detected; Melting: melting temperature.

**Strain**	**Species**	**PCR result**	**LAMP Assay**		**qPCR with TaqMan® Probe (Ct)**	**qPCR with SYBR Green**		**Mycotoxin Production (ppm)**			
			**Tp (min:s)**	**Melting (°C)**		**Ct**	**Melting (°C)**	**AFB1**	**AFB2**	**AFG1**	**AFG2**
CBS 46691	*A. oryzae var. oryzae*	Neg	Neg	Neg	Neg	Neg	Neg	n.d.	n.d.	n.d.	n.d.
CBS 485.65	*A. flavus var. columnaris*	Neg	Neg	Neg	Neg	32.5	78.43	n.d.	n.d.	n.d.	n.d.
CBS 501.65	*A. subolivaceus*	Pos	21:59 ± 03:47	88.97 ± 0.09	26.39 ± 3.82	32.46 ± 0.00	78.58 ± 0.00	n.d.	0.8±0.8	n.d.	n.d.
CBS 120.51	*A. thomii*	Pos	29:27 ± 01:51	87.39 ± 0.09	25.83 ± 1.35	25.62 ± 0.18	78.28 ± 0.21	0.02±0.06	0.6±0.4	n.d.	n.d.
CBS 117610	*A. arachidicola*	Pos	25:30 ± 03:48	89.02 ± 0.09	Neg	Neg	66.07	420.9±293.0	35.1±15.9	1361.1±100.5	543.1±46.5
CBS 117635	*A. minisclerotigenes*	Pos	21:32 ± 01:07	89.12 ± 0.00	16.92 ± 1.32	14.86 ± 0.04	79.25 ± 0.73	34.1±48.6	118.2±239.0	261.8±318.6	n.d.
CBS 121.62	*A. parvisclerotigenus*	Pos	22:08 ± 00:21	89.22 ± 0.09	Neg	Neg	Neg	390.5±46.5	140.6±21.1	639.5±81.0	n.d.

Table S2. Other species used in the specificity panel of the LAMP assay (Tp, time to positive) and the qPCR assay (Ct). The PCR results reported the presence or absence of a 308 bp-amplicon. Standard deviation was included for each Tp and melting temperature. Tp: time to positive. Anneal: melting temperature.

**Strain**	**Species**	**PCR result**	**LAMP Assay**		**qPCR with TaqMan® Probe (Ct)**
			**Tp (min:s)**	**Melting (°C)**	
**COLL1**	*Colletotrichum sp.*	Not tested	Negative	Negative	Negative
**TRICO1**	*Trichoderma sp.*	Not tested	Negative	Negative	Negative
**CBS 115152**	*Alternaria alternata*	Not tested	Negative	Negative	Negative
**CBS 116329**	*Alternaria alternata*	Not tested	Negative	Negative	Negative
**PHC80**	*Phytophthora capsici*	Not tested	Negative	Negative	Negative
**CAL56**	*Penicillium crustosum*	Not tested	Negative	Negative	Negative
**CAL57**	*Penicillium crustosum*	Not tested	Negative	Negative	Negative
**CAL58**	*Penicillium crustosum*	Not tested	Negative	Negative	Negative
**CAL3F**	*Penicillium commune*	Not tested	Negative	Negative	Negative
**SP2**	*Penicillium palitans*	Not tested	Negative	Negative	Negative
**SP3**	*Penicillium palitans*	Not tested	Negative	Negative	Negative
**XF**	*Penicillium solitum*	Not tested	Negative	Negative	Negative
**X3**	*Penicillium polinicum*	Not tested	Negative	Negative	Negative
**X6**	*Penicillium polinicum*	Not tested	Negative	Negative	Negative
**XA**	*Penicillium viridicatum*	Not tested	Negative	Negative	Negative
**XC**	*Penicillium viridicatum*	Not tested	Negative	Negative	Negative
**XB**	*Penicillium yezoense*	Not tested	Negative	Negative	Negative
**CAL66**	*Penicillium glabrum*	Not tested	Negative	Negative	Negative
**CAL67**	*Penicillium glabrum*	Not tested	Negative	Negative	Negative
**XD**	*Penicillium glabrum*	Not tested	Negative	Negative	Negative
**CALA**	*Penicillium citrinum*	Not tested	Negative	Negative	Negative
**CLA1**	*Cladosporium sp.*	Not tested	Negative	Negative	Negative

Table S3. Reference strains used for the identification of the hazelnut isolates. The strains were previously identified by Frisvad et al. [4]. GenBank accession number report the sequence for the calmodulin used to infer a phylogenetic tree that identifies the hazelnut isolates.

Species	Isolate Number	GenBank Accession Number	Species	Isolate Number	GenBank Accession Number
Aspergillus aflatoxiformans	CBS 143679	MG518076	*Aspergillus leporis*	CBS 151.66	MG518033
	CBS 121.62	MG518089	*Aspergillus luteovirescens*	CBS 620.95	MG517998
Aspergillus aliaceus	CBS 536.65	EF661534		CBS 117187	EF661533
	CBS 110.26	MG518004	*Aspergillus minisclerotigenes*	CBS 117635	MG518009
Aspergillus arachidicola	CBS 117610	EF202049		CBS 117633	MG518007
	CBS 117611	MG518006		CBS 117634	MG518008
	CBS 117615	MG517999		DTO 045-F4	MG518021
Aspergillus aspearensis	CBS 143672	MG518040		DTO 045-F5	MG518022
	CBS 143671	MG518038		DTO 045-F6	MG518023
Aspergillus austwickii	CBS 143677	MG518072		DTO 045-I9	MG518024
Aspergillus avenaceus	CBS 109.46	FJ491496		DTO 228-G9	MG518083
	CBS 102.45	FJ491495		DTO 228-H1	MG518084
Aspergillus bertholletius	CBS 143687	JX198674		DTO 228-H5	MG518088
Aspergillus caelatus	CBS 764.97	EF202036	*Aspergillus mottae*	CBS 130016	MG518058
Aspergillus cerealis	CBS 143674	MG518063		MUM 10.233	HM803013
	CBS 143675	MG518064	*Aspergillus neoalliaceus*	CBS 134375	MG518158
Aspergillus coremiiformis	CBS 553.77	FJ491488	*Aspergillus nomius*	CBS 260.88	EF661531
Aspergillus flavus	CBS 100927	EF661508		CBS 399.93	MG518127
	CBS 110.55	MG518005	*Aspergillus novoparasiticus*	CBS 126849	MG518055
	CBS 117637	MG518010		CBS 126850	MG518057
	CBS 117638	MG518011	*Aspergillus oryzae*	CBS 102.07	EF661506
	CBS 118.62	MG517996		NRRL 458	EF661507
	CBS 119368	MG518002	*Aspergillus parasiticus*	CBS 100926	EF661516
	CBS 120.51	MG518012		CBS 104.22	MG517994
	CBS 133263	JX627693		CBS 119.51	MG518000
	CBS 143688	MG518144		CBS 138.52	MG517997
	CBS 143689	MG518145		CBS 260.67	MG518013
	CBS 485.65	MG518014		CBS 580.65	MG518030
	CBS 501.65	MG518015		CBS 822.72	MG518019
	CBS 542.69	MG518016		DTO 203-C4	MG518037
	CBS 574.65	JN185447		DTO 203-H7	MG518043
	DTO 016-I5	MG518003		NRRL 4123	EF661518
	DTO 062-C7	MG517985		DTO 258-D4	MG518097
	DTO 062-C8	MG517986		DTO 283-C6	MG518117
	DTO 062-H7	MG517987		DTO 285-G9	MG518120
	DTO 066-C3	MG517989		NRRL 6433	EF661519
	DTO 087-A4	MG517992		DTO 303-C2	MG518129
	DTO 215-E5	MG518050			
	DTO 258-C9	MG518095			
	DTO 276-H7	MG518104	*Aspergillus pipericola*	CBS 143680	MG518087
	DTO 276-H8	MG518105	*Aspergillus pseudocaelatus*	CBS 117616	MG517995
	DTO 276-H9	MG518106	*Aspergillus pseudonomius*	CBS 119388	EF661529
	DTO 276-I1	MG518107		DTO 177-G7	MG518029
	DTO 276-I3	MG518109	*Aspergillus pseudotamarii*	CBS 766.97	EF661521
	DTO 276-I4	MG518110		CBS 765.97	EF661520
	DTO 276-I5	MG518111	*Aspergillus sergii*	CBS 130017	MG518059
	DTO 276-I6	MG518112	*Aspergillus sojae*	CBS 100928	EF202041
	DTO 276-I7	MG518113		CBS 133.52	EF661517
	DTO 276-I8	MG518114		DTO 173-C3	MG518028
	DTO 281-E2	MG518115	*Aspergillus subflavus*	CBS 143683	MG518143
	DTO 281-H8	MG518116		S843b	MG518164
	DTO 285-F6	MG518118	*Aspergillus tamarii*	CBS 104.13	EF661526
	DTO 285-I4	MG518123		CBS 133097	MG518049

	DTO 300-C7	MG518124		CBS 133393	EU021686
	DTO 300-D7	MG518125	***Aspergillus togoensis***	CBS 205.75	FJ491489
	NRRL 20521	EF661514	***Aspergillus transmontanensis***	CBS 130015	HM803020
	NRRL 3518	EF661510		MUM 10.205	HM803021
	NRRL 4822	EF661513	***Aspergillus vandermerwei***	CBS 612.78	EF661540
Aspergillus lanosus	CBS 650.74	MG518017		CBS 129201	MG518032
